# Biotransformation of bisphenol A analogues by the biphenyl-degrading bacterium *Cupriavidusbasilensis* - a structure-biotransformation relationship

**DOI:** 10.1007/s00253-020-10406-4

**Published:** 2020-03-03

**Authors:** Marie-Katherin Zühlke, Rabea Schlüter, Annett Mikolasch, Ann-Kristin Henning, Martin Giersberg, Michael Lalk, Gotthard Kunze, Thomas Schweder, Tim Urich, Frieder Schauer

**Affiliations:** 1 Institute of Microbiology, University of Greifswald, Felix-Hausdorff-Straße 8, 17489 Greifswald, Germany; 2 Institute of Pharmacy, University of Greifswald, Felix-Hausdorff-Straße 3, 17489 Greifswald, Germany; 3 Institute of Marine Biotechnology, Walter-Rathenau-Straße 49a, 17489 Greifswald, Germany; 4 Leibniz Institute of Plant Genetics and Crop Plant Research (IPK), Corrensstraße 3, OT Gatersleben, 06466 Seeland, Germany; 5 Institute of Biochemistry, University of Greifswald, Felix-Hausdorff-Straße 4, 17489 Greifswald, Germany

**Keywords:** Biodegradation, Degradation, Metabolism, Bacteria, Micropollutants, Estrogenic activity

## Abstract

Comparative analyses determined the relationship between the structure of bisphenol A (BPA) as well as of seven bisphenol analogues (bisphenol B (BPB), bisphenol C (BPC), bisphenol E (BPE), bisphenol F (BPF), bisphenol Z (BPZ), bisphenol AP (BPAP), bisphenol PH (BPPH)) and their biotransformability by the biphenyl-degrading bacterium *Cupriavidus basilensis* SBUG 290*.* All bisphenols were substrates for bacterial transformation with conversion rates ranging from 6 to 98% within 216 h and 36 different metabolites were characterized. Transformation by biphenyl-grown cells comprised four different pathways: (a) formation of *ortho-*hydroxylated bisphenols, hydroxylating either one or both phenols of the compounds; (b) ring fission; (c) transamination followed by acetylation or dimerization; and (d) oxidation of ring substituents, such as methyl groups and aromatic ring systems, present on the 3-position. However, the microbial attack of bisphenols by *C. basilensis* was limited to the phenol rings and its substituents, while substituents on the carbon bridge connecting the rings were not oxidized. All bisphenol analogues with modifications at the carbon bridge could be oxidized up to ring cleavage, while substituents at the 3-position of the phenol ring other than hydroxyl groups did not allow this reaction. Replacing one methyl group at the carbon bridge of BPA by a hydrophobic aromatic or alicyclic ring system inhibited both dimerization and transamination followed by acetylation. While most of the bisphenol analogues exhibited estrogenic activity, four biotransformation products tested were not estrogenically active.

**Electronic supplementary material:**

The online version of this article (10.1007/s00253-020-10406-4) contains supplementary material, which is available to authorized users.

## Introduction

For more than six decades, bisphenol A (BPA) has been used in the production of polycarbonate plastics and epoxy resins (EPA 2014; Hoekstra and Simoneau 2013; Usman and Ahmad 2016) with an annual production of more than 3.8 million tons (Michałowicz 2014). It is one of the most extensively used bisphenols with applications in thermal paper production (EPA 2014; Pivnenko et al. 2015), food contact material, electronic devices, water pipes, or health care (Michałowicz 2014; Vandenberg et al. 2007). There are many studies pointing to environmental contamination resulting in unavoidable human exposure, with risks particularly associated with its potential to act as endocrine agent (Usman and Ahmad 2016). In reaction to public concern, some countries, especially in North America and the European Union, regulated the production and restricted the usage of BPA (Barroso 2011; Goldinger et al. 2015). As a result, chemicals with similar structure, referred to as bisphenol analogues, were introduced for industrial applications to replace BPA (Chen et al. 2016). For example, in thermal paper production, 19 different bisphenol analogues have been documented (EPA 2014). Bisphenol analogues include, among others, bisphenol AP (BPAP), bisphenol B (BPB), bisphenol C (BPC), bisphenol E (BPE), bisphenol F (BPF), bisphenol PH (BPPH), and bisphenol Z (BPZ). All of these have in the meantime found widespread applications. BPAP, for example, is used in polymer materials and in the fine chemical industry (Zhang et al. 2013a). BPF is applied in the manufacture of fire-resistant polymers (Delfosse et al. 2012) and used in lacquers, varnishes, liners, adhesives plastics, and water pipes (Fromme et al. 2002). The increasing usage of bisphenol analogues raised questions about their environmental fate, degradability, and endocrine activity. Many of them have been documented in various environmental compartments like indoor dust, sediment, sludge, and surface waters, in foods and food containers, personal care products, as well as in human samples as comprehensively summarized by Chen et al. (2016). Many of these substances constitute serious public health risks. Bisphenol analogues, such as BPE or BPF, show estrogenic activity similar to that of BPA while BPB or BPZ are even more active (Kojima et al. 2019). BPF is known to exhibit genotoxicity (Cabaton et al. 2009) and oxidative toxicity (Michałowicz et al. 2015). It also enhances reactive oxygen species formation, increases lipid peroxidation, and changes the activities of superoxide dismutase, catalase, and glutathione peroxidase in human erythrocytes (Macczak et al. 2017). Even though there is an increasing number of studies regarding the estimation of human exposure, uptake and health risks, toxicity, estrogenicity, and environmental fate of these bisphenol analogues, the knowledge currently available is not sufficient to assess the impact of these compounds on human health and the environment. Chen et al. (2016) pointed out the serious lack of information regarding environmental persistence, toxicity, and elucidation of metabolic pathways and products of bisphenol analogues. With the present study, we narrow some of these gaps by investigating the biotransformation of seven bisphenol analogues by the biphenyl-degrading bacterium *Cupriavidus basilensis* SBUG 290 that was isolated from environmental samples and is able to transform BPA efficiently (Zühlke et al. 2017). Our objectives were (i) to determine the biotransformation pathway of the various compounds by structure elucidation of the transformation products formed, (ii) to establish principles of the structure-biotransformation-relationship, and (iii) to determine the estrogenicity of the transformation products compared to that of the bisphenol analogues.

## Materials and methods

### Strain

The bacterial strain was isolated from compost soil by enrichment cultures with 4-chlorobiphenyl (Becher et al. 2000) and later identified by 16S rRNA analyses as *C. basilensis* (Zühlke et al. 2017). The bacterial strain is deposited in the strain collection of the Department of Biology of the University of Greifswald (SBUG) as *C. basilensis* SBUG 290.

### Biotransformation experiments

2,2-Bis-(4-hydroxy-3-methylphenyl)-propane (bisphenol C, BPC), 1,1-bis-(4-hydroxyphenyl)ethane (bisphenol E, BPE), 1,1-bis-(4-hydroxyphenyl)methane (bisphenol F, BPF), and 4,4′-cyclohexylidenebisphenol (bisphenol Z, BPZ) were purchased from Sigma-Aldrich (Steinheim, Germany), and 1,1-bis-(4-hydroxyphenyl)-1-phenylethane (bisphenol AP, BPAP), 2,2-bis-(4-hydroxyphenyl)butane (bisphenol B, BPB), and 2,2-bis(2-hydroxy-5-biphenylyl)propane (bisphenol PH, BPPH) were purchased from Tokyo Chemical Industry GmbH Co (Tokyo, Japan), at highest purity available. Cells of *C. basilensis* were cultivated for 8 h in nutrient broth (NB) and afterwards for an additional 72 h with biphenyl as substrate as described earlier (Zühlke et al. 2017). For biotransformation experiments, 500-ml-flasks containing 100 ml mineral salts medium for bacteria (MMb; pH 6.3; Hundt et al. 1998; Stope et al. 2002) were used. The medium was supplemented with 0.002% (20 mg L^−1^) or 0.006% (60 mg L^−1^) of the bisphenols, each applied from 5% stock solutions in dimethylformamide. After 72 h of cultivation with biphenyl, cells were harvested by centrifugation (15,890×*g*, 25 min and 4 °C), washed twice in MMb and finally resuspended in a small amount of MMb. This cell suspension was transferred to the medium supplemented with the bisphenols until an optical density (OD_500 nm_) of 3.0 was reached. This corresponds to approximate 5.2 × 10^9^ cells mL^−1^. Cells were incubated on a rotary shaker at 30 °C and 180 rpm for 216 h (HT FORS). Flasks with (i) MMb and the respective bisphenol in the absence of cells and (ii) with MMb without bisphenols but with cells (OD_500 nm_ = 3.0) served as controls. Biotransformation experiments were carried out in independent duplicate experiments.

### Analytical methods for detection of bisphenols and transformation products

For monitoring the transformation of the bisphenol analogues and the formation of transformation products, samples of 1 mL of cell suspension were removed periodically: the first time directly after cell transfer to MMb with bisphenols (this corresponds to 0 h) and then each or every second day until 216 h of incubation. Cells were pelleted by centrifugation (3587×*g*, 5 min, room temperature), and the cell-free supernatant was analyzed by high-performance liquid chromatography (HPLC) using an Agilent Technologies 1200 Series system (Santa Clara, USA). Components were separated on a LiChroCART® 125-4 RP-18 endcapped 5 μm (Merck, Darmstadt, Germany) column applying a solvent system of methanol and phosphoric acid (0.1%, v/v) with a linear gradient from 30 to 100% methanol over a period of 14 min at a flow rate of 1 mL min^−1^. A diode array detector was used for signal recording. Extraction and product isolation were carried out according to Zühlke et al. (2017). On this basis, the supernatant of 10 to 20 centrifuged cultures (corresponding to 1 to 2 L volume) was extracted and the products were isolated as described for the products I-IV of BPA transformation (Zühlke et al. 2017). Usually, a bisphenol concentration of 0.006% was used to yield the highest product concentrations. This did not apply for BPZ where cells were exposed to 0.002% of substrate and for BPF where product IV_BPF_ was only detected after applying 0.002% BPF.

### Analytical methods for structure elucidation of transformation products

High-performance liquid chromatography-mass spectrometry (HPLC-MS) and gas chromatography-mass spectrometry (GC-MS) were performed on the equipment and with the separation conditions described earlier (Zühlke et al. 2017). Nuclear magnetic resonance (NMR) spectra of all products were recorded on a Bruker Avance-II instrument (Bruker Biospin GmbH, Rheinstetten, Germany) at 600.27 MHz (^1^H-NMR) and 150.1 MHz (^13^C-NMR) in MeOH-d_4_.

### Determination of estrogenicity of bisphenols and transformation products

To determine estrogenic properties of certain transformation products and the bisphenol analogues from which they were formed, the *Arxula adeninivorans* estrogen screen kit (A-YES assay) from new_diagnostics GmbH (Freising, Germany) was used as described (Zühlke et al. 2016). Estrogenicity was determined at least in duplicate.

## Results

**Fig. 1:**
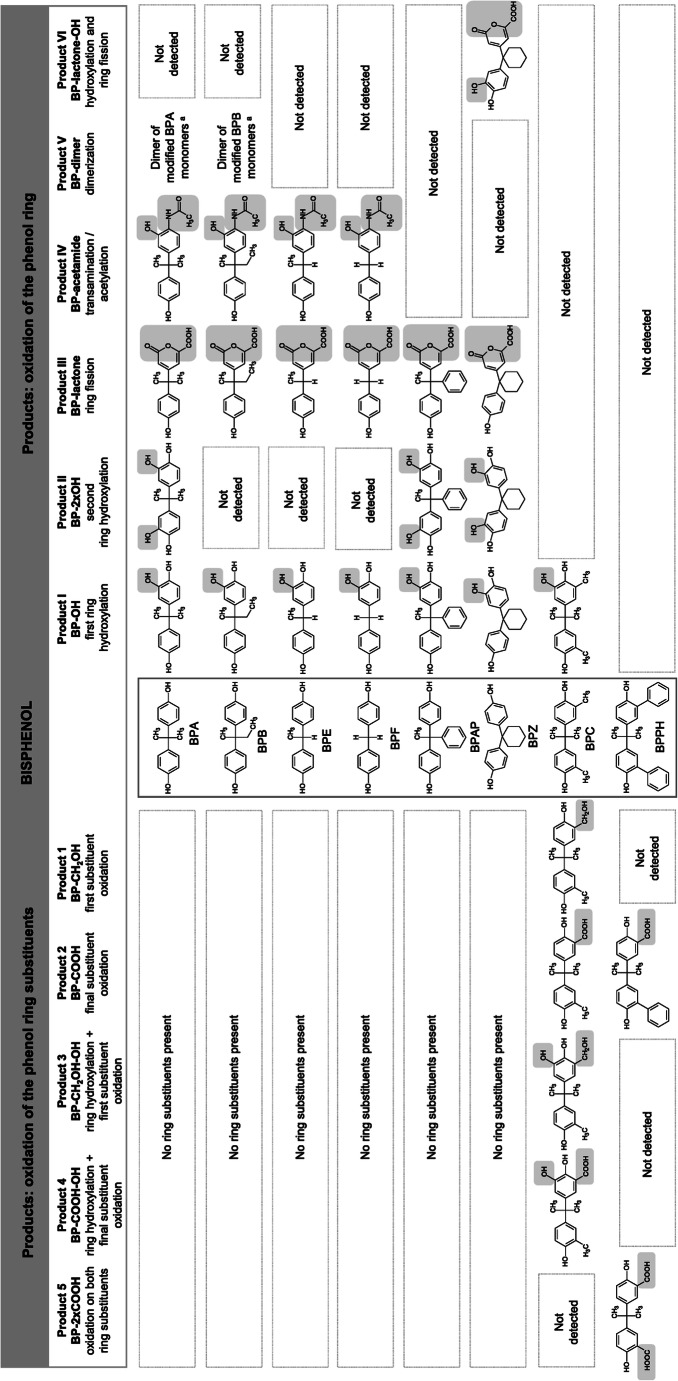
Overview of detected and characterized products, with corresponding abbreviations used in the text, formed during the incubation of *Cupriavidus basilensis* SBUG 290 with eight different bisphenols. Products of BPA transformation refer to Zühlke et al. (2017). ^a^Dimer consisting of modified bisphenol-monomers with at least one *ortho*-quinonimine, an *ortho*-quinoid and an unmodified ring

In the present study, we investigated the biotransformation of seven BPA analogues, which differ from BPA by the addition or by the lack of substituents at the ring linking carbon bridge (as with BPAP, BPB, BPE, BPF, and BPZ) or at the phenol rings (as with BPC and BPPH). For the chemical structures, see middle column of Fig. 1.

### Transformation rates of bisphenol A analogues

Biphenyl-grown cells of *C. basilensis* were able to transform all of these compounds but could not use them as substrates for growth. Using a concentration of 0.006%, transformation rates ranged between 6 and 98% within 216 h of incubation: BPC (98%) > BPB (62%) > BPE (31%) > BPF (6%), see Supplementary Figs. S1-S5. Rates for transformation of BPAP, BPZ, and BPPH were not determined due to their low solubility in the medium (Supplementary Figs. S3 and S5). By comparison, 85% of BPA was transformed using the same incubation conditions.

### Transformation of bisphenol analogues to products—principle and overview

During transformation of seven bisphenol A analogues, 36 different metabolites were characterized biochemically and the structures for 24 of these products were identified (Supplementary Figs. S1-S5). HPLC analysis provided initial information on the structure of the products formed, which were then named according to the already characterized transformation products of BPA transformation (Zühlke et al. 2017). Additional abbreviations were used to indicate the structure of products, e.g., BP-OH for the *ortho-*hydroxylated product I (Fig. 1) or BPB-OH if the respective bisphenol is specified. Structure elucidation was based on HPLC, HPLC-MS, GS-MS, and/or NMR analysis, as well as comparison with already identified products.

### Identification of transformation products of bisphenol analogues with unsubstituted phenol rings and different substituents at the carbon bridge

*C. basilensis* converted bisphenols with unsubstituted phenol rings and different substituents at the carbon bridge to three (BPAP, BPE, BPF) or up to four (BPB, BPZ) transformation products (Fig. 1). Analytical data of the transformation products were very similar to data on the characterized products of BPA transformation (Zühlke et al. 2017).

#### Identification of one-ring *ortho*-hydroxylated products (designated as products I; BP-OH)

During the incubation of *C. basilensis* with BPAP, BPB, BPE, BPF, and BPZ for 216 h, one product each was detected by HPLC analysis. These products have a similar UV-Vis spectrum compared to that of product I_BPA_ (Zühlke et al. 2017), with two absorption maxima at around 220 and 280 nm (Table 1). By analogy, the products were named product I with subscript bisphenol analogue abbreviations. These products were extracted at pH 7 and they eluted 1.0 to 1.4 min earlier from the RP-18-column than did their parent compounds, suggesting a more hydrophilic character. Using mass spectrometry analyses, the mass differences of *m*/*z* 16 between the products and the parent compounds (Supplementary Table S1) indicated the introduction of a hydroxyl group. GC-MS analyses did not detect products I in each case, most probably due to low product amounts, or to insufficient methylation. However, for product I of BPA, BPAP, BPE, and BPZ transformation, the detection of mono-, di-, and/or trimethylated derivatives of the products confirmed the presence of an additional hydroxyl group, resulting in a trihydroxylated molecule. A second hydroxyl group in *ortho*-position to the phenolic hydroxyl group was verified by NMR analyses for product I_BPE_ (Supplementary Table S2). Thus, it is postulated that *C. basilensis ortho*-hydroxylated one phenol ring of bisphenols (Fig. 1).

**Table 1:**
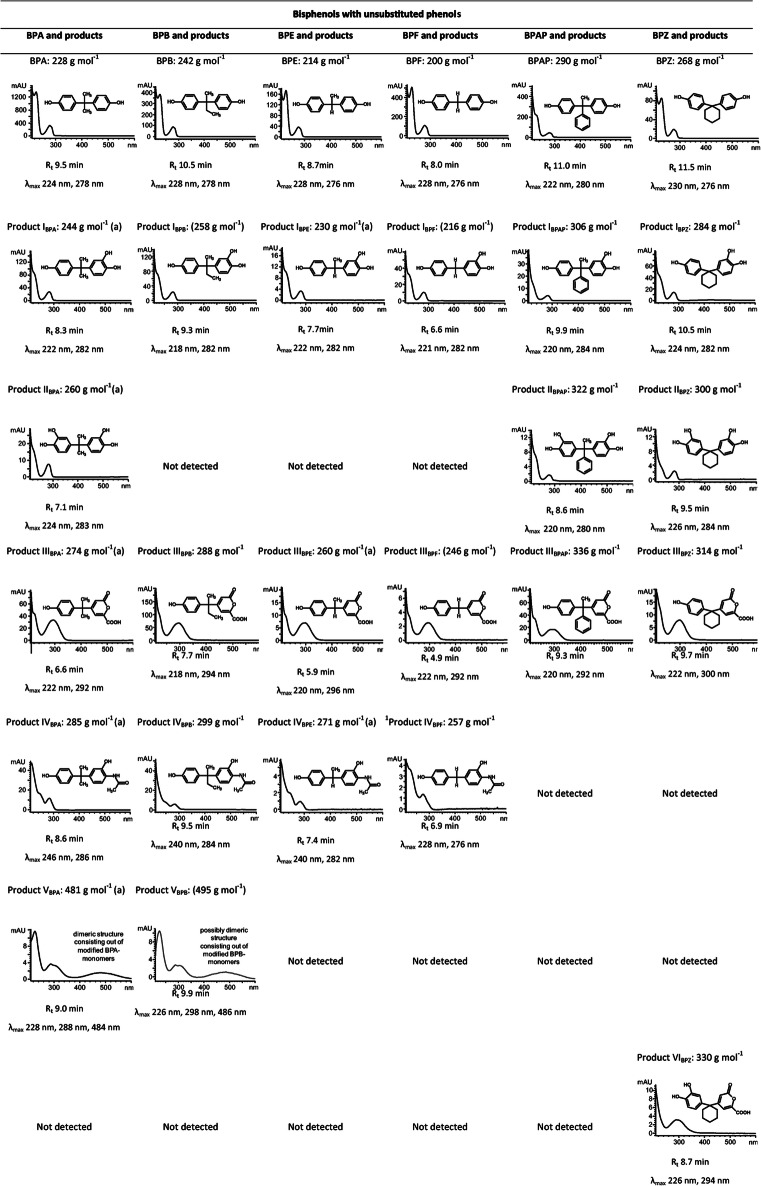
UV-Vis spectra, retention time (*R*_t_), absorption maxima (*λ*_max_) of BPA (according to Zühlke et al. 2017), BPB, BPE, BPF, BPAP, BPZ, and of products formed during the incubation of *Cupriavidus basilensis* SBUG 290 with the individual bisphenols as well as proposed structure and molecular mass

Proposed structure and derived molecular mass based on comparison with elucidated products of BPA (Zühlke et al. 2017) and usually confirmed by HPLC, HPLC-MS, and/or GC-MS data (see Supplementary Information); if masses are marked with an “(a)” additional NMR-data are available; note that molecular masses in brackets represent theoretical masses as only HPLC-data were generated; products without any suggestions concerning structure are listed in Supplementary Table S10 (Supplementary Information)

#### Identification of products *ortho*-hydroxylated at both phenol rings (designated as products II; BP-2xOH)

HPLC analyses of the culture supernatant of *C. basilensis* incubated with BPAP and BPZ revealed one product each with UV-Vis spectra having two maxima, one at around 220 nm and the other at around 280 nm (Table 1). Both the UV-Vis spectra and the shift in the retention time at the RP-18-column compared to the substrates (about 2 min earlier) showed strong similarity to the data of product II_BPA_ (Zühlke et al. 2017). Both products were extracted at pH 7. The GC-MS data showed a mass difference of *m*/*z* 32 between the products and the respective bisphenol analogues and pointed to another hydroxyl group on the aromatic ring system in addition to products I (Supplementary Table S3). Furthermore, both products II were detected in the HPLC-MS negative ion mode only (Supplementary Table S3) as was product II_BPA_. Because of the similarity of the HPLC, GC-MS, and HPLC-MS data of products II with corresponding data of product II_BPA_, whose structure was confirmed by NMR analyses (Zühlke et al. 2017), a structure of products *ortho*-hydroxylated on both phenol rings was postulated for product II_BPAP_ and product II_BPZ_ (Fig. 1).

#### Identification of ring fission products with lactone structure (designated as products III; BP-lactone)

During incubation of *C. basilensis* with BPAP, BPB, BPE, BPF, and BPZ, one product each eluted about 2–3 min earlier than the substrates from the RP-18-column. The UV-Vis spectra of the products with two absorption maxima at around 220 and 290–300 nm (Table 1) corresponded to that of product III_BPA_ (Zühlke et al. 2017), and these products are therefore designated as products III with subscript bisphenol analogue abbreviations. HPLC-MS analyses of the products III, all present in the extract at pH 2, revealed a higher mass peak for the underivatized products III in comparison to the respective bisphenol analogues with a mass difference of *m*/*z* 46 (Supplementary Table S4). GC-MS analyses did not detect products III in each case, most probably due to low yield or insufficient methylation. However, for product III of BPB, BPE, and BPZ transformation, the detection of a mono- and dimethylated derivative of the respective product confirmed the presence of two groups, which can be methylated. Based on NMR analyses of product III_BPA_ (Zühlke et al. 2017) and product III_BPE_ (Supplementary Table S5), all products III could be identified as ring fission products with lactone structure (Fig. 1).

#### Identification of *ortho*-hydroxylated products with an acetamide substituent (designated as products IV; BP-acetamide)

After incubation of *C. basilensis* with BPB, BPE, and BPF, products IV were detected by HPLC analyses, which showed absorption maxima at around 230–240 and 280 nm and thus were similar to the ones of product IV_BPA_ (Zühlke et al. 2017). These products also had reduced retention times (∆ 0.9–1.3 min) compared to the bisphenol analogues (Table 1). The difference of *m*/*z* 57 between the products IV, extracted at pH 7, and the bisphenol analogues (Supplementary Table S6) strongly hinted at an acetamide substituent as shown for product IV_BPA_ (Zühlke et al. 2017). NMR analyses of product IV_BPE_ (Supplementary Table S7) confirmed this structure. Thus, all products IV were postulated to be *ortho*-hydroxylated products with an acetamide substituent (Fig. 1).

#### Identification of dimers of modified bisphenol monomers (designated as products V; BP-dimer)

HPLC analysis of the culture supernatant during the incubation of *C. basilensis* with BPB revealed one product, named product V_BPB_, with a retention time shift similar to product V_BPA_ (Zühlke et al. 2017). The similarity of the UV-Vis spectrum with absorption maxima at 226, 298, and 486 nm (Table 1) with that of product V_BPA_, which was postulated to be a dimer consisting of an *ortho*-quinonimine, an *ortho*-quinoid and an unmodified ring and further structures, suggests a similar overall structure for product V_BPB_. However, final structure elucidation was not possible by the methods available.

#### Identification of ring fission products with lactone structure hydroxylated on the remaining phenol ring (designated as products VI; BP-lactone-OH)

One product was only detected by HPLC analysis during the incubation of *C. basilensis* with BPZ and named product VI_BPZ_. The UV-Vis spectrum was similar to that of the ring fission product III_BPZ_, but product VI_BPZ_ eluted 1 min earlier from the RP-18-column (Table 1). HPLC-MS analysis revealed a base ion peak at *m*/*z* 331 (positive ion mode) and *m*/*z* 329 (negative ion mode). The molecular mass of *m*/*z* 330 and the difference of *m*/*z* 16 between product VI_BPZ_ and product III_BPZ_ (Supplementary Table S8) pointed to a hydroxylated ring fission product with lactone structure. GC-MS analysis also detected one trimethylated derivative of product VI_BPZ_ confirming the presence of an additional hydroxyl group. These structural data led to the identification of product VI_BPZ_ as 4-[1-(3,4-dihydroxyphenyl)-cyclohexyl]-6-oxo-6H-pyran-2-carboxylic acid, a ring fission product with lactone structure, *ortho*-hydroxylated on the uncleaved phenol ring (Fig. 1).

Postulated structures and chemical names of all products identified are summarized in Supplementary Table S9. Products detected by HPLC but without any further information as to their structure are listed in Supplementary Table S10.

### Identification of transformation products of bisphenol analogues with substituted phenol rings

*C. basilensis* converted BPC (*ortho*-substituted with a methyl group at each phenol ring) to five major products (Fig. 1; Supplementary Fig. S4). BPPH (*ortho*-substituted with an aromatic ring system at each phenol ring) was transformed to two major products (Supplementary Fig. S5), and another seven minor products were formed in very low amounts (Supplementary Table S10). Because of the low yields, these were not further characterized.

#### Identification of product I_BPC_ (BPC-OH)

The UV-Vis spectrum of product I_BPC_ was similar to that of product I_BPA_ (Zühlke et al. 2017) and all other products I, with absorption maxima at 224 and 282 nm, and a retention time reduced by about 1 min compared to the parent compound (Table 2). These data pointed to a trihydroxylated derivative. HPLC, GC-MS (Supplementary Table S11), and NMR analyses (Supplementary Table S12) led to the proposed structure 5-[1-(4-hydroxy-3-methyl-phenyl)-1-methyl-ethyl]-3-methyl-benzene-1,2-diol for product I_BPC_, a product *ortho*-hydroxylated at one phenol ring (Fig. 1).

**Table 2:**
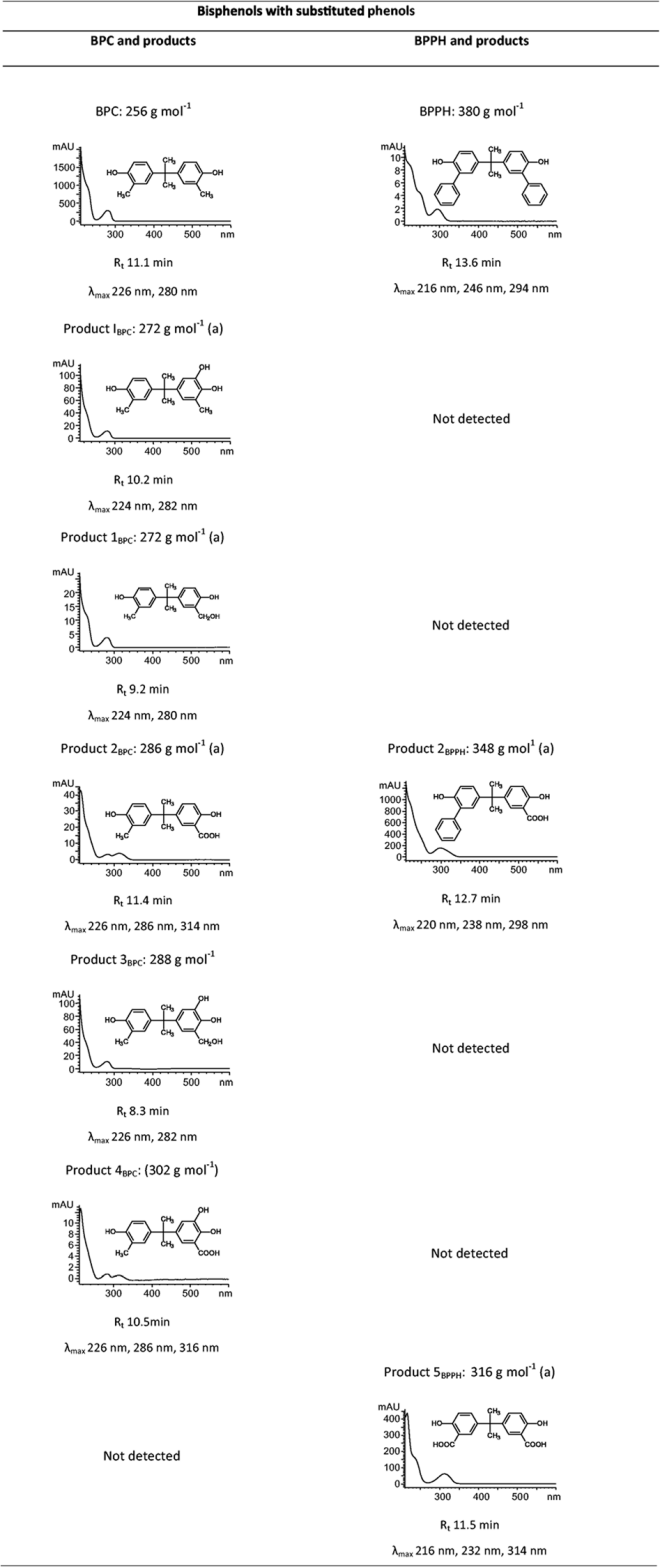
UV-Vis spectra, retention time (*R*_t_), absorption maxima (*λ*_max_) of BPC and BPPH and of products formed during the incubation of *Cupriavidus basilensis* SBUG 290 with the individual bisphenols as well as proposed structure and molecular mass

Proposed structure and derived molecular mass based on comparison with elucidated products of BPA (Zühlke et al. 2017) and usually confirmed by HPLC, HPLC-MS, and/or GC-MS data (see Supplementary Information); if masses are marked with an “(a)” additional NMR-data are available; note that molecular masses in brackets represent theoretical masses as only HPLC-data were generated; products without any suggestions concerning structure are listed in Supplementary Table S10 (Supplementary Information)

#### Identification of product 1_BPC_ (BPC-CH_2_OH)

In contrast to product I_BPC_, where one phenol ring was hydroxylated, GC-MS and NMR analyses of product 1_BPC_ pointed to an additional substituent at the methyl substituent of one phenol ring (Supplementary Tables S11-S13). The presence of no additional proton signals to those of BPC in the aromatic range and an additional methylene signal at 4.6 ppm in the ^1^H NMR spectrum and at 61.4 ppm in the ^13^C NMR spectrum led to the identification of product 1_BPC_ as 2-(hydroxymethyl)-4-[1-(4-hydroxy-3-methyl-phenyl)-1-methyl-ethyl]-phenol (Fig. 1).

#### Identification of product 2_BPC_ (BPC-COOH)

Product 2_BPC_ was analyzed by HPLC-UV-Vis, HPLC-MS, GC-MS, and NMR. The UV-Vis spectrum has three maxima at 226, 286, and 314 nm (Table 2) and is readily distinguishable from the spectra of the substrate and all other products, but it is very similar to the spectrum of product 4_BPC_ (see data below). The NMR analyses of product 2_BPC_ showed the presence of only one aromatic methyl substituent (^1^H 2.12 ppm, ^13^C 16.5 ppm), and one aromatic carboxyl group (^13^C 174.3 ppm) formed from the second aromatic methyl substituent (Supplementary Tables S11, S14). All other NMR signals are comparable with those of the substrate. These data led to the description of product 2_BPC_ as 2-hydroxy-5-[1-(4-hydroxy-3-methyl-phenyl)-1-methyl-ethyl]benzoic acid (Fig. 1).

#### Identification of product 3_BPC_ (BPC-CH_2_OH-OH)

HPLC analysis and the UV-Vis spectrum of product 3_BPC_ indicated an additional *ortho*-hydroxylation of the aromatic ring of product 1_BPC_ (Table 2). Experiments determined a retention time shift of Δ1.9 min between BPC and product 1_BPC_. A similar retention time shift was seen between product I_BPC_ and product 3_BPC_. Whereas a Δ1.9 min was thus indicative for the oxidation of the methyl group to CH_2_OH, a retention time shift of Δ0.9 min was characteristic for ring-*ortho*-hydroxylation between BPC and product I_BPC_, as well as between product 1_BPC_ and product 3_BPC_ (Table 2). HPLC and HPLC-MS (Supplementary Table S11) analyses led to the proposed structure of 3-(hydroxymethyl)-5-[1-(4-hydroxy-3-methyl-phenyl)-1-methyl-ethyl]benzene-1,2-diol for product 3_BPC_, a one-ring *ortho*-hydroxylated product with an additional hydroxylated methyl substituent at the same ring (Fig. 1).

#### Identification of product 4_BPC_ (BPC-COOH-OH)

Because of low yield, product 4_BPC_ was only characterized by HPLC-UV-Vis and by comparison of these data with those of product 2_BPC_. The UV-Vis spectrum of product 4_BPC_ has three maxima at 226, 286, and 316 nm (Table 2) and is very similar to that of product 2_BPC_ (BPC-COOH, see data above). The HPLC retention time shift of product 2_BPC_ (BPC-COOH) compared to that of product 4_BPC_ (BPC-COOH-OH) is the same as the HPLC retention time shift of product 1_BPC_ (BPC-CH_2_OH) compared to that of product 3_BPC_ (BPC-CH_2_OH-OH). Both are Δ0.9 min, indicating an additional hydroxyl group (see data above). Furthermore, the time shift of product 2_BPC_ (BPC-COOH) compared to that of product 1_BPC_ (BPC-CH_2_OH) is the same as the time shift of product 4_BPC_ (BPC-COOH-OH) compared to that of product 3_BPC_ (BPC-CH_2_OH-OH). Both are Δ 2.2 min, pointing to an oxidation of the hydroxymethyl group to a carboxyl group. This indicated that one methyl group of BPC was oxidized to a carboxyl group and one hydroxy group was introduced into the same aromatic ring. All data indicate comparable structure patterns for product 4_BPC_ and product 2_BPC_ so that product 4_BPC_ can be described as 2,3-dihydroxy-5-[1-(4-hydroxy-3-methyl-phenyl)-1-methyl-ethyl]benzoic acid (Fig. 1).

#### Identification of product 2_BPPH_ (BPPH-COOH)

The structure of product 2_BPPH_ was analyzed by comparing the NMR and MS data with those of product 5_BPPH_ (see below), and product 2_BPC_ of BPC transformation, and the parent compound (Supplementary Tables S14-S18). The very complex NMR data of product 2_BPPH_ showed the presence of one carboxyl group at the aromatic ring similar to that of product 5_BPPH_ and product 2_BPC_. Furthermore, the signals of two additional aromatic rings similar to those in the substrate BPPH were detected. The HPLC-MS data (Supplementary Table S18) also support the structure of product 2_BPPH_ as 2-hydroxy-5-[1-(4-hydroxy-3-phenyl-phenyl)-1-methyl-ethyl]benzoic acid (Fig. 1).

#### Identification of product 5_BPPH_ (BPPH-2xCOOH)

Before considering product 2_BPPH_, we first determined the structure of product 5_BPPH_, because of its more easily identifiable NMR signals. The NMR data of product 5_BPPH_ are very similar to those for the aromatic ring with the carboxyl group of product 2_BPC_ (Supplementary Tables S14, S17), indicating the cleavage of both substituted phenyl rings and further degradation to carboxyl groups. The HPLC-MS data (Supplementary Table S18) fit this proposed structure, and therefore, product 5_BPPH_ can be described as 5-[1-(3-carboxy-4-hydroxy-phenyl)-1-methyl-ethyl]-2-hydroxy-benzoic acid (Fig. 1).

### Kinetics of product formation

Measured amounts of one-ring *ortho*-hydroxylated products I (BP-OH) reached their respective maximum within 24–48 h of incubation with BPB, BPC, BPE, and BPF. This was considerably postponed for products I of BPAP and BPZ. In all cases, concentrations of products I then decreased again. Similar kinetics were determined for product 1_BPC_ (BPC-CH_2_OH) of BPC transformation, where a methyl substituent of one phenol ring was oxidized, and for product II_BPZ_ (BPZ-2xOH) of BPZ transformation, where both phenol rings are *ortho*-hydroxylated. The other products, e.g., products III (BP-lactone) or products IV (BP-acetamide) accumulated in the supernatant (Supplementary Figs. S1-S5).

### Estrogenicity of some products and bisphenols

**Fig. 2:**
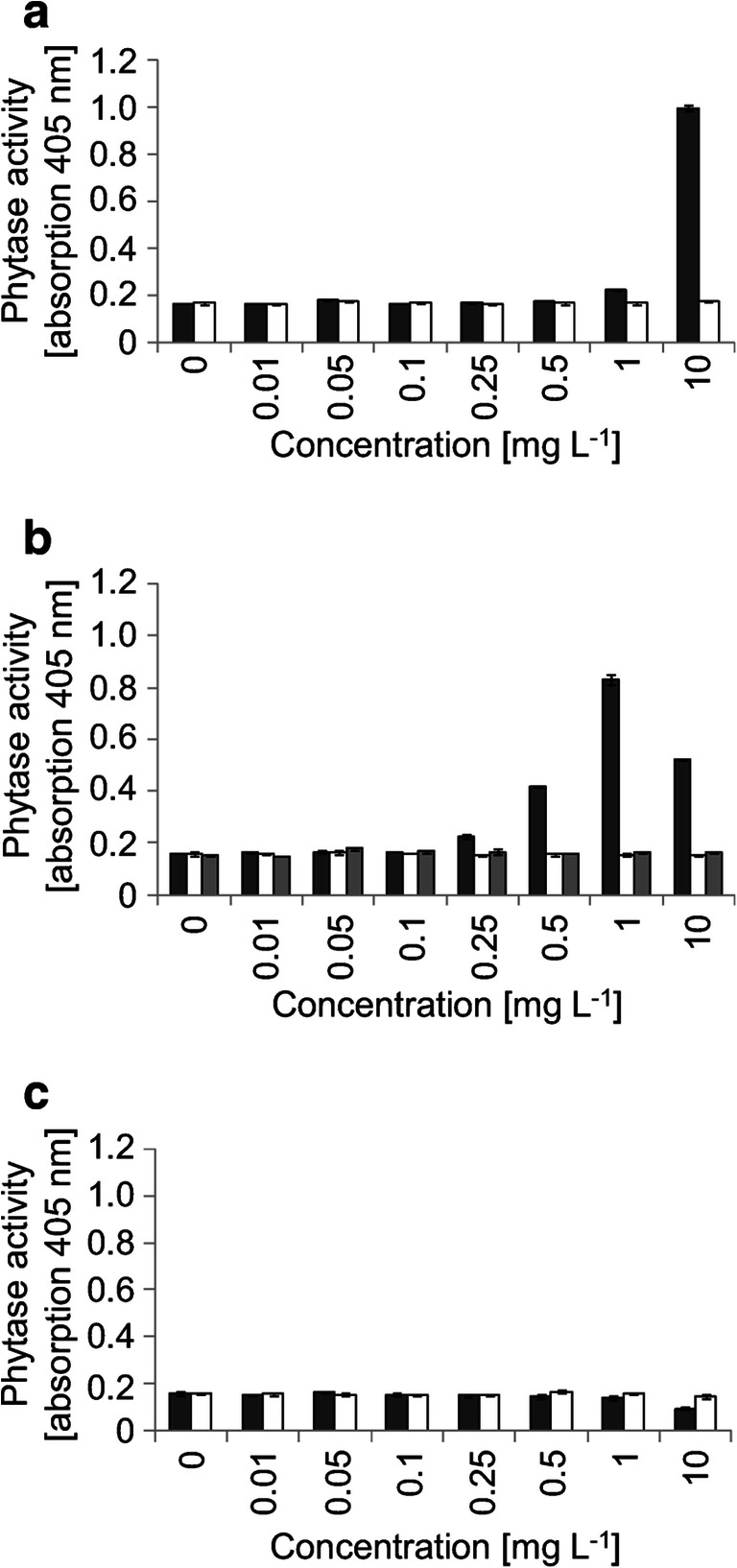
Reporter gene activity (phytase) of the transgenic yeast *Arxula adeninivorans* at different concentrations of **a** product I_BPE_ (BPE-OH; open bars) compared to BPE (dark gray–filled bars), **b** product 1_BPC_ (BPC-CH_2_OH; light gray–filled bars) and product 3_BPC_ (BPC-CH_2_OH-OH; open bars) compared to BPC (dark gray–filled bars), and **c** product 2_BPPH_ (BPPH-COOH; open bars) compared to BPPH (dark gray–filled bars)

The A-YES-assay was used to determine the estrogenic activity of selected parent compounds and transformation products. In this assay, the human estrogen receptor expressed in yeast drives a phytase gene whose activity can be followed spectrophotometrically. While BPE induced phytase activity at a concentration of 1 mg L^−1^ (Fig. 2a) and BPC at 0.25 mg L^−1^ and are thus estrogenically active, phytase activity was not induced by BPPH (Fig. 2c). Those bisphenol-derived products that were formed by *C. basilensis* in sufficient amounts for purification were isolated and analyzed for their estrogenic properties. Neither product I_BPE_ (BPE-OH) of BPE, product 1_BPC_ (BPC-CH_2_OH) and product 3_BPC_ (BPC-CH_2_OH-OH) of BPC nor product 2_BPPH_ (BPPH-COOH) of BPPH exhibited estrogenic activities (Fig. 2a–c).

## Discussion

In this study, we extended previous investigations on the transformation of BPA by *C. basilensis* SBUG 290 (Zühlke et al. 2017) to five bisphenols with unsubstituted phenol rings (BPB, BPE, BPF, BPAP, and BPZ) and two with ring substituents (BPC and BPPH). These bisphenols all now have widespread industrial applications.

For relatively water-soluble bisphenols, biotransformation rates within 9 days (216 h) were determined by HPLC (Supplementary Figs. S1-S5). The extent of transformation by *C. basilensis* ranged from 98 to 6% (BPC > BPA > BPB > BPE > BPF). Data comparing microbial removal of bisphenols are scarce, and they are in contrast to our findings. For example, in the Gram-positive bacterium *Arthrobacter* sp., the removal efficiency was BPF > BPA, although the final degradation extents of BPF and BPA were rather similar (Ren et al. 2016). A similar order (BPA, BPB, BPF, BPS > BPE > BPC) was determined for biodegradation of bisphenols by the 4-*tert*-butylphenol utilizing *Sphingobium fuliginis* OMI (Ogata et al. 2012). In wastewater treatment plants (with mixed culture conditions), the order was BPAP > BPP > BPF > BPZ > BPC > BPS > BPB > BPA > BPE > BPAF (Wang et al. 2019). In contrast, Sun et al. (2017) estimated BPS > BPA > BPF in a wastewater treatment plant, while BPAP and BPE were persistent. In this latter case, besides biodegradation, also bioadsorption was taken into account as a modulating factor of these values. Bisphenols in river sediments were ranked by their biodegradability under aerobic conditions BPF >> BPA > BPC > BPB >> BPS (Ike et al. 2006), confirmed by Chang et al. (2014) with BPF > BPA > BPB. In contrast, using anaerobic conditions, the BPS degradation rate was clearly enhanced: BPF > BPS, BPA > BPE > BPB (Ike et al. 2006). Thus, at least, the very low conversion rate of BPF by *C. basilensis* does not correspond to the values estimated for microbial populations in rivers and wastewater. On the other hand, BPC and BPB removal is relatively efficient in *C. basilensis* SBUG 290. Doubtless, the removal rate of bisphenols will vary with the type of microorganism involved, whether the cells are growing or non-growing, the type of degradation pathway (primary attack at the aromatic ring or at the carbon bridge), the conditions of incubation (single species, e.g., in labs, or mixed culture, e.g., in waste water treatment plants), and the substrate concentration available.

**Fig. 3:**
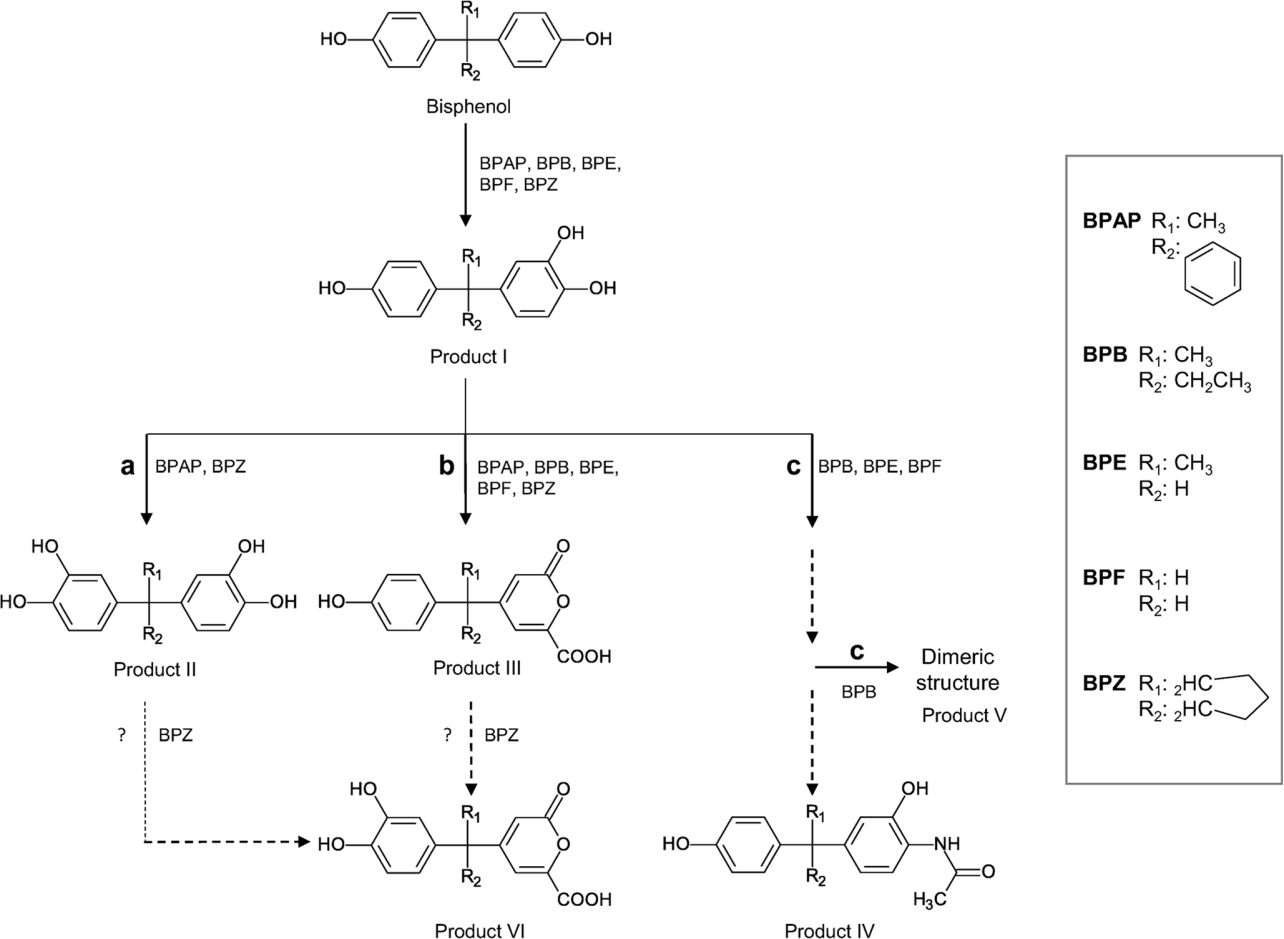
Proposed pathway for the biotransformation of BPAP, BPB, BPE, BPF, and BPZ by *Cupriavidus basilensis* SBUG 290 via product I by (a) a second *ortho*-hydroxylation, (b) ring fission and combination of a second *ortho*-hydroxylation and ring fission, as well as (c) transamination followed by acetylation or dimerization

Whereas the extents of biotransformation of the different bisphenol analogues by *C. basilensis* diverge, we found that the transformation mechanisms of those bisphenols with unsubstituted phenol rings but with varying substituents at the ring linking carbon bridge (BPB, BPE, BPF, BPAP, BPZ) are similar to the mode of BPA transformation. *C. basilensis* initially hydroxylated one phenol ring of these bisphenols in the *ortho*-position. The corresponding one-ring *ortho*-hydroxylated intermediates were substrates for (a) *ortho*-hydroxylation of the other phenol ring, (b) ring cleavage, and/or (c) transamination followed by acetylation or dimer formation (Fig. 3). All bisphenols with unsubstituted phenol rings were hydroxylated at one phenol ring, while intermediates hydroxylated at both phenol rings were detected for BPAP and BPZ only. In contrast to *C. basilensis*, the 4-*tert*-butylphenol utilizing *S. fuliginis* OMI is able to *ortho*-hydroxylate both phenol rings in BPB, BPE, BPC, and BPS (Ogata et al. 2012). Corresponding products of BPA were also formed by an undefined soil microbial consortium (Choi and Lee 2017).

Likewise, all five bisphenols with unsubstituted phenol rings were subjected to ring cleavage analogously to the transformation of BPA by *C. basilensis*, resulting in products with lactone structure. Kinetics of product formation (Supplementary Fig. S3) indicated that the compound hydroxylated at both phenol rings was an additional substrate for ring cleavage only in the case of BPZ, but not for BPAP. Hydroxylation and subsequent cleavage of one phenol ring of bisphenols by *C. basilensis* SBUG 290 serve for detoxification (Zühlke et al. 2017), which has also been reported for corresponding products of *p*-*tert*-amylphenol transformation by this strain (Schlueter et al. 2014). This is due to the fact that *C. basilensis* SBUG 290 is not able to grow with these compounds, just as *S. fuliginis* OMI (Ogata et al. 2012), a strain able to cleave the aromatic ring system of BPA resulting in formation of monoaromatic compounds, is unable to grow on BPA. Aerobic soil biodegradation of BPA, BPAF, and BPS via ring cleavage has also been postulated (Choi and Lee 2017).

The third pathway - transamination of a one-ring *ortho*-hydroxylated intermediate followed by acetylation - was shown only for BPB, BPE, and BPF. The formation of these products was inhibited when the carbon bridge was substituted with an aromatic (BPAP) or alicyclic (BPZ) ring system or when the phenol rings were substituted. The formation of dimers of modified bisphenol monomers, in analogy to BPA, was only detected in the case of BPB.

All bisphenols with unsubstituted phenol rings differ in the substituents at the carbon bridge. Irrespective of the nature of these substituents, *C. basilensis* SBUG 290 could neither oxidize them nor cleave the bridge and thus could not use the bisphenols as carbon and energy source. This is in contrast to the situation with *C. basilensis* JF1 (Fischer et al. 2010), which introduces oxygen into the molecule followed by cleavage into 4-(2-propanol)-phenol and *p*-hydroquinone. Other bacterial strains that target the carbon bridge are often able to degrade bisphenols. A rearrangement of the bridge enabled cleavage of bisphenols (BPA) into monocyclic aromatic hydrocarbons as reported for strain MV1 (Lobos et al. 1992; Spivack et al. 1994), *Sphingomonas bisphenolicum* AO1, *Sphingomonas* sp. TTNP3 (Kolvenbach et al. 2007; Zhang et al. 2013b) or *Shewanella haliotis* MH137742 (de Santana et al. 2019).

**Fig. 4:**
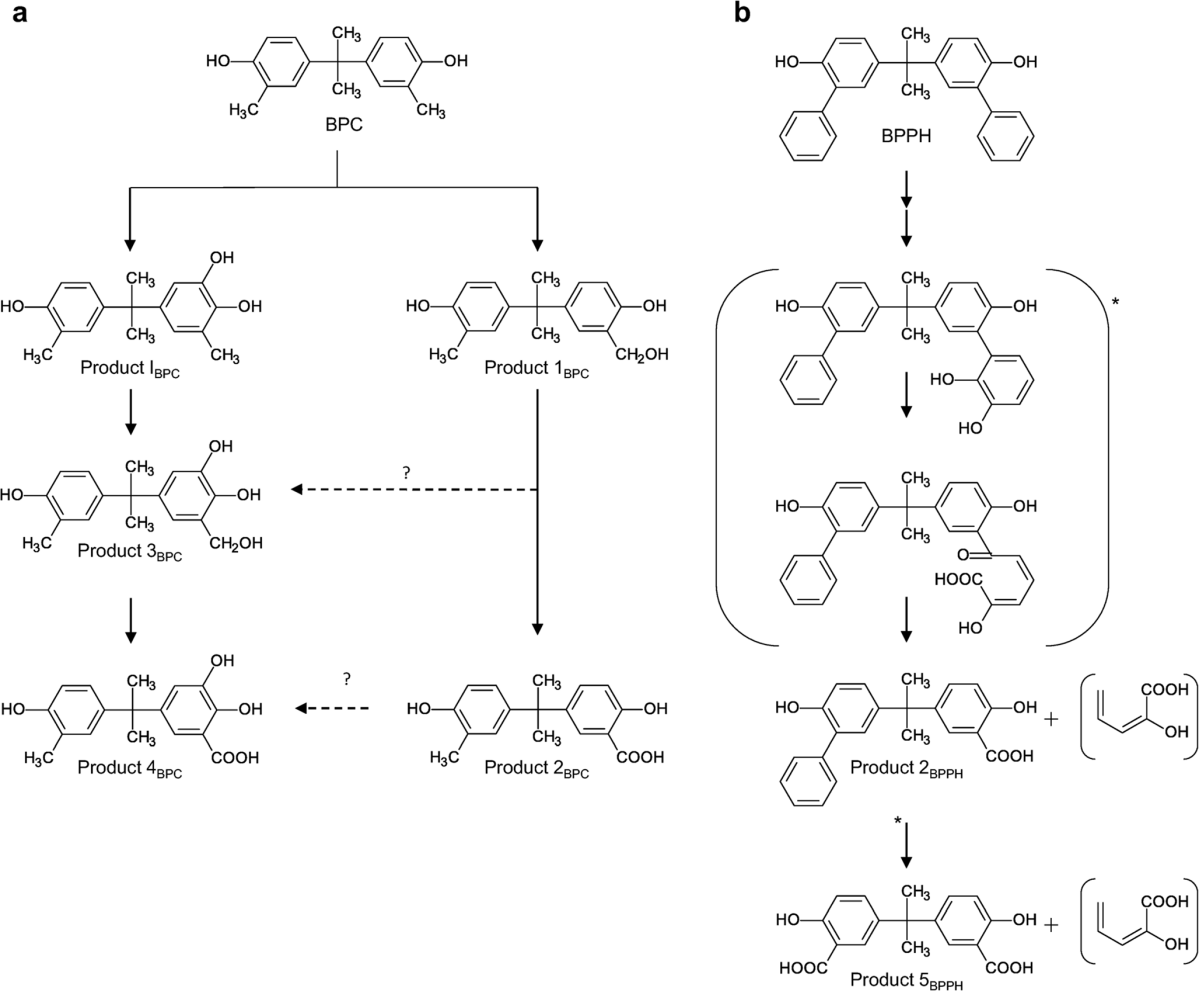
Proposed pathway for the biotransformation of **a** BPC and **b** BPPH by *Cupriavidus basilensis* SBUG 290

Bisphenols with substituted phenol rings were substrates for novel transformation reactions compared to the biotransformation of BPA by *C. basilensis* SBUG 290. BPC und BPPH are characterized by additional *ortho*-substituents at their phenol rings. These substituents prevented ring cleavage, transamination followed by acetylation and dimerization, but at the same time served as additional targets for bacterial transformation. When an aromatic ring system is connected to the phenol ring, resulting in a biphenyl-like structure (BPPH), biphenyl-grown cells of *C. basilensis* SBUG 290 can cleave this substituted ring and oxidize it up to a carboxyl group (Fig. 4), probably in the same manner as biphenyl (Wesche et al. 2005). When the phenol ring is substituted with a methyl group in the *ortho*-position (BPC), one phenol ring of BPC was not only *ortho*-hydroxylated, but the methyl group was also oxidized (Fig. 4), as reported here for the first time. A corresponding reaction was observed for the anaerobic bacterium *Castellaniella defragrans*, where a limonene dehydrogenase hydroxylated the methyl group of cyclic monoterpenes (Puentes-Cala et al. 2018). Fungi hydroxylate methyl groups at alicyclic ring systems, too (Schlüter et al. 2019). In *S. bisphenolicum* AO1, a cytochrome P450 monooxygenase catalyzed hydroxylation of methyl substituents at the BPA carbon bridge (Sasaki et al. 2005a, b, 2008). This was not detected for *C. basilensis* SBUG 290, where oxidation of the methyl groups is restricted to the substituents at the phenol rings. However, another P450 system that oxygenates thiocarbamate herbicides was characterized in a *Cupriavidus* species (*C. metallidurans:* De Mot and Parret 2002; Warman et al. 2012), but neither its occurrence in *C. basilensis* nor its substrate selectivity is hitherto known.

Another mechanism for bisphenol transformation is the formation of conjugates. The formation of BPA glucosides has been shown in *Aspergillus fumigatus* (Yim et al. 2003), in *Cunninghamella elegans* (Keum et al. 2009) and in plants (Morohoshi et al. 2003; Nakajima et al. 2007), and BPA glucuronides were detected in rats (Inoue et al. 2001). Bacteria can form conjugates as well. While *Bacillus amyloliquefaciens* (Zühlke et al. 2016) converts various bisphenols into phosphate conjugates as a detoxification mechanism, no conjugates were detected in *C. basilensis* SBUG 290.

**Fig. 5:**
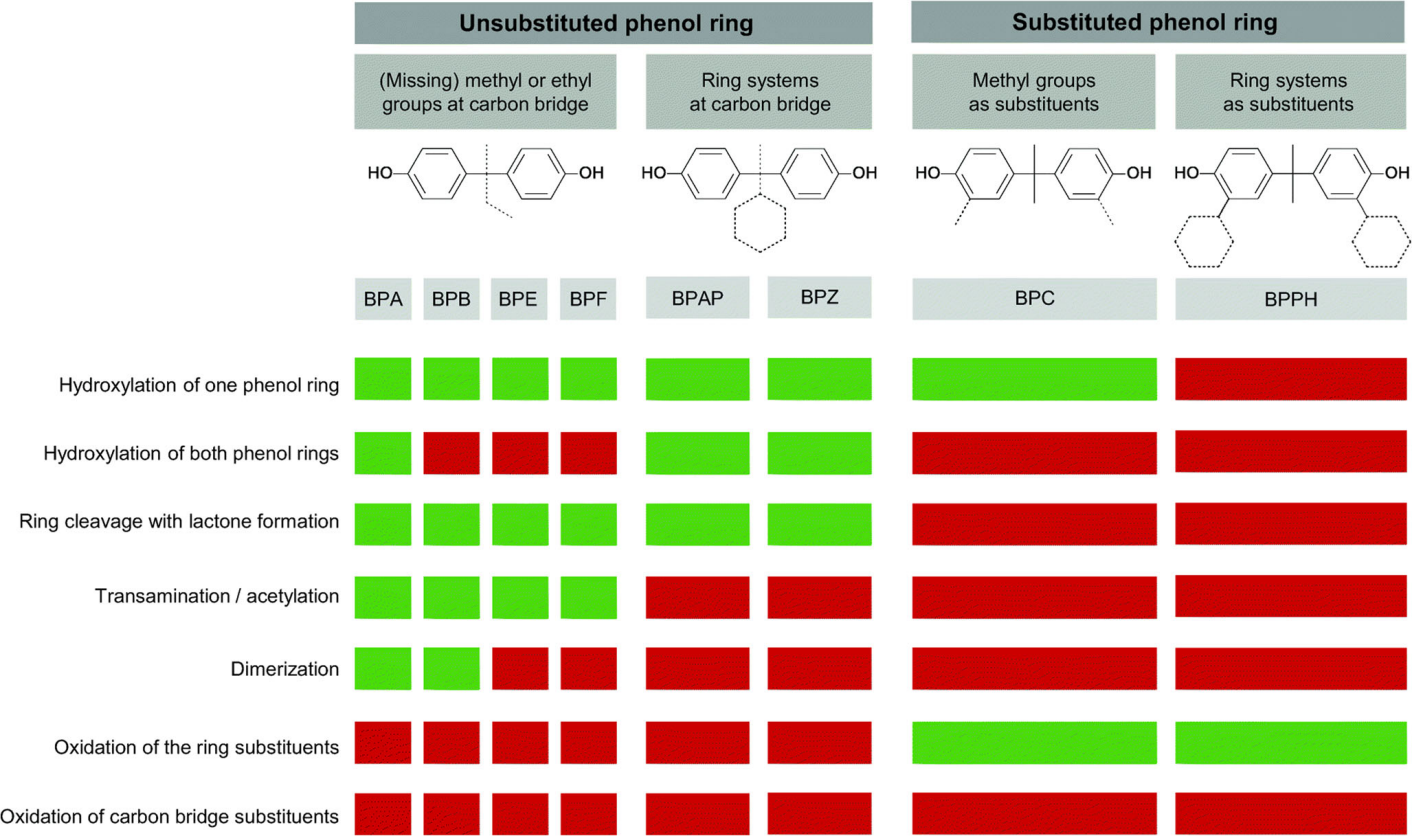
Structure-biotransformation-relationship of bisphenols used as substrates for bacterial transformation by *Cupriavidus basilensis* SBUG 290. Different bisphenols are shown as simplified structures. Dashed lines indicate substituents, which vary in case of bisphenols with unsubstituted phenols (Fig. 1). Green: transformation reaction detected; red: transformation reaction not detected

When bisphenols enter the microbial cell, but cannot be used as carbon and energy source, the cells need to detoxify these hazardous compounds. As a result, certain structure-biotransformation relationships become apparent (Fig. 5). All bisphenols with unsubstituted phenol rings are substrate for (a) hydroxylation and (b) ring cleavage with lactone formation (Fig. 5). When the carbon bridge is substituted with small ligands like methyl and ethyl groups and/or hydrogen, (c) transamination followed by acetylation was carried out, too. Larger substituents, like aromatic or alicyclic rings, prevented this pathway. Microbial dimerization of transformation products seems to be possible only when methyl and/or ethyl groups are present at the carbon bridge. Bisphenols with *ortho*-substituted phenol rings are not substrates either for ring cleavage or for transamination followed by acetylation or dimerization. Thus, the basic structure of the bisphenol molecule itself is, with one exception (ring-hydroxylation of BPC), not modified, and only (d) the substituents at the phenolic ring were oxidized.

Depending upon the structure of the bisphenols, *C. basilensis* SBUG 290 formed 5 products with BPA and 36 products with 7 bisphenol analogues as parent compounds. This may reflect what happens in the environment where a consortium of microorganisms may catalyze various reactions. Because of this, quantification of pollutants should also include important metabolites or derivatives as well as their toxic and endocrine activities. In terms of estrogenic activity, BPC is more estrogenic active than BPE and the non-estrogenic BPPH, while the transformation products of BPC, BPE, and BPPH tested did not exhibit estrogenic activity. Estrogenicity of bisphenols themselves correlates with their hydrophobicity, making BPAP, BPZ, BPB, or BPC more estrogenic than BPA and BPE or BPF (Kitamura et al. 2005; Kojima et al. 2019; Zühlke et al. 2016). Despite being very hydrophobic, BPPH is a larger molecule and might thus mask important coactivator regions (Heldring et al. 2007). This may also apply to its products. In contrast, the reduced estrogenic activity of the hydroxylated products of BPC and BPE might be due to reduced hydrophobicity resulting in a weaker affinity towards the hydrophobic ligand binding side of the ERα receptor (Coleman et al. 2003; Gao et al. 1999), which corresponds to previous results (Kitamura et al. 2005; Skledar and Masic 2016). In addition, lactone formation as well as transamination followed by acetylation may also lead to reduced endocrine activity as confirmed for the corresponding products of BPA transformation by *C. basilensis* SBUG 290 (Zühlke et al. 2017). Thus, *C. basilensis* SBUG 290 has a broad repertoire of transformation mechanisms, accepting not only bisphenols but also biphenyl, 4-chlorobiphenyl, dibenzofuran, 9*H*-carbazol, and *p*-*tert*-amylphenol (Becher et al. 2000; Hundt et al. 1998; Schlueter et al. 2014; Waldau et al. 2009; Wesche et al. 2005). As shown above for bisphenols, *C. basilensis* not only performs single biotransformation steps but also allows versatile transformation cascades for the individual bisphenol analogues leading to more hydrophilic products with decreased estrogenicity. Compared to reversible conjugate formation (Gonzalez-Gil et al. 2019; Zühlke et al. 2016), these products might be stable or serve as substrates for further degradation by other microorganisms in the environment.

## Electronic supplementary material

ESM 1 (PDF 969 kb).
