# Correction to: Biotransformation of bisphenol a analogues by the biphenyl-degrading bacterium *Cupriavidus basilensis* - a structure-biotransformation relationship

**DOI:** 10.1007/s00253-021-11421-9

**Published:** 2021-07-16

**Authors:** Marie-Katherin Zühlke, Rabea Schlüter, Annett Mikolasch, Ann-Kristin Henning, Martin Giersberg, Michael Lalk, Gotthard Kunze, Thomas Schweder, Tim Urich, Frieder Schauer

**Affiliations:** 1 Institute of Microbiology, University of Greifswald, Felix-Hausdorff-Straße 8, 17489 Greifswald, Germany; 2 Institute of Pharmacy, University of Greifswald, Felix-Hausdorff-Straße 3, 17489 Greifswald, Germany; 3 Institute of Marine Biotechnology, Walter-Rathenau-Straße 49a, 17489 Greifswald, Germany; 4 Leibniz Institute of Plant Genetics and Crop Plant Research (IPK), Corrensstraße 3, OT Gatersleben, 06466 Seeland, Germany; 5 Institute of Biochemistry, University of Greifswald, Felix-Hausdorff-Straße 4, 17489 Greifswald, Germany

**Correction to: Applied Microbiology and Biotechnology (2020) 104:3569–3583**

10.1007/s00253-020-10406-4

The article **“Biotransformation of bisphenol A analogues by the biphenyl-degrading bacterium** ***Cupriavidus basilensis*** **- a structure-biotransformation relationship”**, written by Marie-Katherin Zühlke, Rabea Schlüter, Annett Mikolasch, Ann-Kristin Henning, Martin Giersberg, Michael Lalk, Gotthard Kunze, Thomas Schweder, Tim Urich and Frieder Schauer, was originally published Online First without open access. After publication in volume 104, issue 8, page 3569–3583, the author decided to opt for Open Choice and to make the article an open access publication. Therefore, the copyright of the article has been changed to © The Author(s) 2020 and the article is forthwith distributed under the terms of the Creative Commons Attribution 4.0 International License (http://creativecommons.org/licenses/by/4.0/), which permits use, duplication, adaptation, distribution and reproduction in any medium or format, as long as you give appropriate credit to the original author(s) and the source, provide a link to the Creative Commons license, and indicate if changes were made. The images or other third party material in this article are included in the article’s Creative Commons license, unless indicated otherwise in a credit line to the material. If material is not included in the article’s Creative Commons license and your intended use is not permitted by statutory regulation or exceeds the permitted use, you will need to obtain permission directly from the copyright holder. To view a copy of this license, visit http://creativecommons.org/licenses/by/4.0.Open access funding enabled and organized by Projekt DEAL. Universität Greifswald.

The original article was corrected.

