# Supplementary material for: Biotransformation of bisphenol A analogues by the biphenyl-degrading bacterium Cupriavidusbasilensis - a structure-biotransformation relationship
Source: Appl Microbiol Biotechnol. 2020 Mar 3;104(8):3569–83. doi: 10.1007/s00253-020-10406-4 (PMC8282568; doi:10.1007/s00253-020-10406-4)
Supplement: Supplementary file 1 — (PDF 969 kb). [file 253_2020_10406_MOESM1_ESM.pdf]

Journal: Applied Microbiology and Biotechnology

Biotransformation of bisphenols by the biphenyl-degrading bacterium *Cupriavidus basilensis* – A structure-biotransformation relationship

Marie-Katherin Zühlke[1,2,3], Rabea Schlüter[1], Annett Mikolasch[1], Ann-Kristin Henning[1], Martin Giersberg[4], Michael Lalk[5], Gotthard Kunze[4], Thomas Schweder[2,3], Tim Urich[1], Frieder Schauer[1]

[1]Institute of Microbiology, University of Greifswald, Felix-Hausdorff-Straße 8, 17489 Greifswald, Germany
[2]Institute of Pharmacy, University of Greifswald, Felix-Hausdorff-Straße 3, 17489 Greifswald, Germany
[3]Institute of Marine Biotechnology, Walter-Rathenau-Straße 49a, 17489 Greifswald, Germany
[4]Leibniz Institute of Plant Genetics and Crop Plant Research (IPK), Corrensstraße 3, OT Gatersleben, 06466 Seeland, Germany
[5]Institute of Biochemistry, University of Greifswald, Felix-Hausdorff-Straße 4, 17489 Greifswald, Germany

Corresponding author: Rabea Schlüter, University of Greifswald, Institute of Microbiology, Felix-Hausdorff-Straße 8, 17489 Greifswald. Phone: +49-3834-864292. Fax: +49-3834-864202. e-mail: rabea.schlueter@uni-greifswald.de

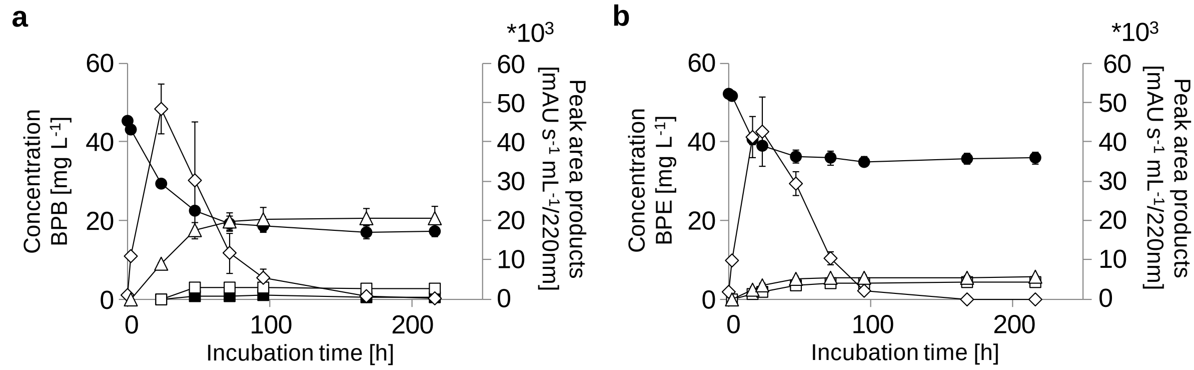

Fig. S1: Biotransformation of 0.006 % (a) BPB (●) and (b) BPE (●) as well as formation of corresponding product I (BP-OH;◇), product III (BP-lactone;△), product IV (BP-acetamide;□) and product V (BP-dimer;■) by *Cupriavidus basilensis* SBUG 290. Structures of bisphenols and their corresponding products are given in Table S9 (page 15/16)

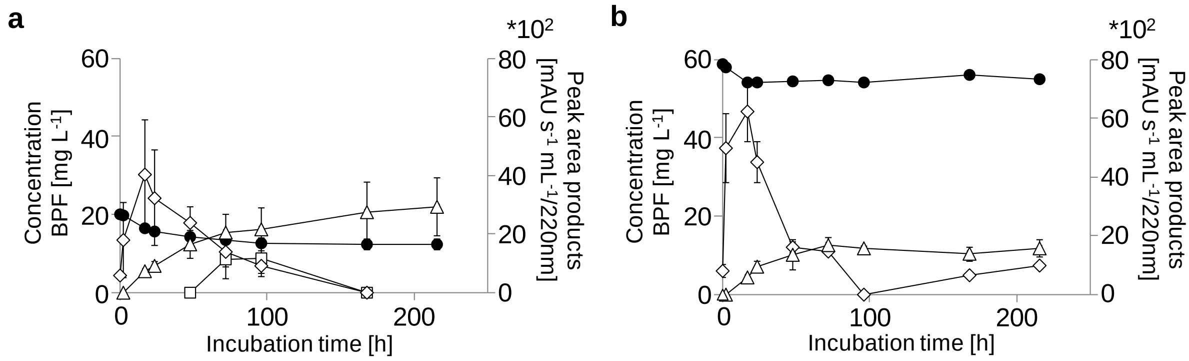

Fig. S2: Biotransformation of (a) 0.002 % BPF (●) and (b) 0.006 % BPF (●) as well as formation of corresponding product I_{BPF} (BPF-OH;◇), product III_{BPF} (BPF-lactone;△) and/or product IV_{BPF} (BPF-acetamide;□) by *Cupriavidus basilensis* SBUG 290. In this case, kinetics for both concentrations of BPF are depicted as product IV was only detected using 0.002 % BPF. Structures of BPF and its corresponding products are given in Table S9 (page 15/16)

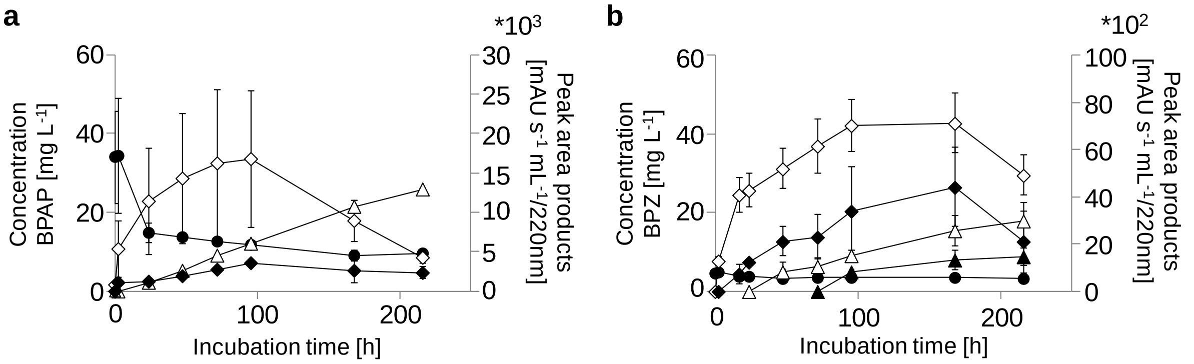

Fig. S3: Biotransformation of (a) 0.006 % BPAP (●) and (b) 0.002 % BPZ (●) as well as formation of corresponding product I (BP-OH;◇), product II (BP-2xOH;◆), product III (BP-lactone;△) and/or product VI (BP-lactone-OH;▲) by *Cupriavidus basilensis* SBUG 290. Structures of bisphenols and their corresponding products are given in Table S 9. Note that the detected bisphenol concentration is much lower than the applied concentration due to low solubility of these bisphenols in the medium

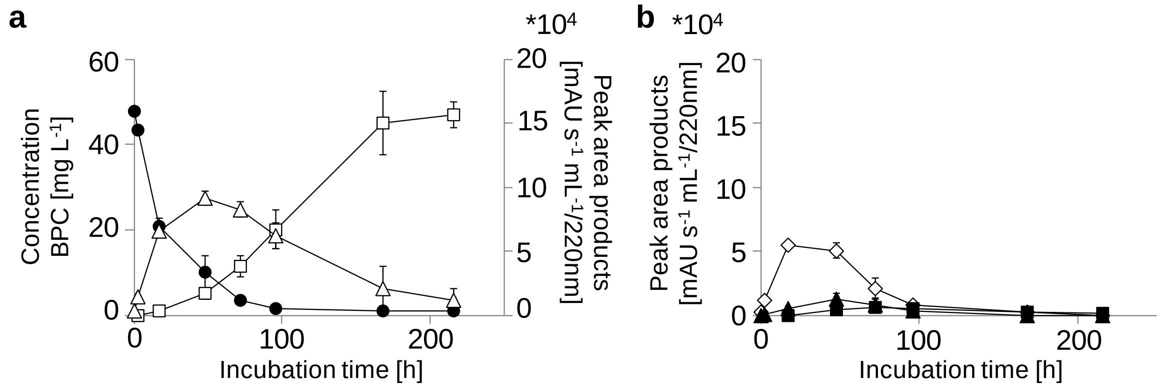

Fig. S4: Biotransformation of (a) 0.006 % BPC (●) and formation of product 1_{BPC} (BPC-CH_2OH;△) and product 2_{BPC} (BPC-COOH;□) as well as (b) product I_{BPC} (BPC-OH;◇), product 3_{BPC} (BPC-CH_2OH-OH;▲) and product 4_{BPC} (BPC-COOH-OH;■) by *Cupriavidus basilensis* SBUG 290. Structure of BPC and its corresponding products are given in Table S9 (page 15/16)

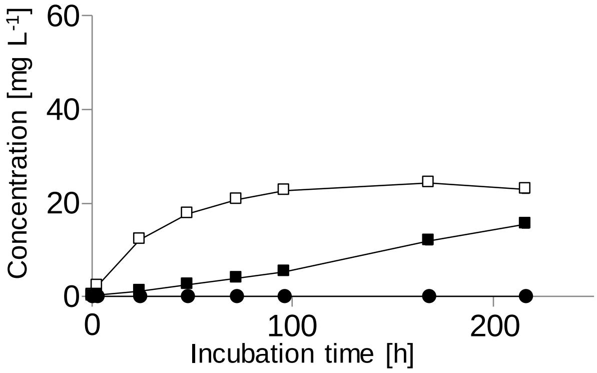

Fig. S5: Biotransformation of 0.002 % BPPH (●) and formation of product 2_{BPPH} (BPPH-COOH;□) and product 5_{BPPH} (BPPH-2xCOOH;■) by *Cupriavidus basilensis* SBUG 290. Structure of BPPH and its corresponding products are given in Table S9 (page 15/16). Note that the detected bisphenol concentration is much lower than the applied concentration due to low solubility of BPPH in the medium

Table S1: GC-MS and HPLC-MS data of bisphenol analogues and their *ortho*-hydroxylated products I (BP-OH) formed during the incubation of *Cupriavidus basilensis* SBUG 290 with BPAP, BPB, BPC, BPE, BPF, and BPZ in comparison to the corresponding product I_{BPA} of BPA-transformation (Zühlke et al. 2017). Chemical structures and names of products are given in Table S9 (page 15/16)

Bisphenol	Molecular weight (g moL^{-1})	Product	Theoretical molecular weight (g moL^{-1})	Mass difference between bisphenol and transformation product	HPLC-MS data		Base peak ion (GC-MS) and if applicable number of methylated derivatives[a]
					negative ion mode $[M-H]^-$	positive ion mode $[M+H]^+$	
BPA	228	product I_{BPA}	244	Δ16 g moL^{-1}	*m/z* 243	n.d.	3x *m/z* 258 (M^++1CH_3)<br>3x *m/z* 272 (M^++2CH_3)<br>1x *m/z* 286 (M^++3CH_3)
BPAP	290	product I_{BPAP}	306	Δ16 g moL^{-1}	*m/z* 305	n.d.	306 (M^+)
BPB	242	product I_{BPB}	258	Δ16 g moL^{-1}	n.d.	n.d.	n.d.
BPE	214	product I_{BPE}	230	Δ16 g moL^{-1}	*m/z* 229	n.d.	230 (M^+)<br>2x *m/z* 244 (M^++1CH_3)<br>1x *m/z* 258 (M^++2CH_3)
BPF	200	product I_{BPF}	216	Δ16 g moL^{-1}	n.d.	n.d.	n.d.
BPZ	268	product I_{BPZ}	284	Δ16 g moL^{-1}	*m/z* 283	n.d.	2x *m/z* 298 (M^++1CH_3)<br>3x *m/z* 312 (M^++2CH_3)<br>1x *m/z* 326 (M^++3CH_3)

n.d. not detected; [a]M^++1CH_3 monomethylated-, M^++2CH_3 dimethylated- or M^++3CH_3 trimethylated-derivative, 1-3x refers to number of detected derivatized substances: three hydroxyl groups within the molecule (see Table S 9) would allow up to three methylations and different combinations thereof to be detected by GC-MS

Table S2: NMR data and structure of product I_{BPE} (BPE-OH) formed during the incubation of *Cupriavidus basilensis* SBUG 290 with BPE

^1H	^1H-^{13}C-correlation	^{13}C	structure
1.50, d, J = 7.2 Hz, 3H, H-8	44.6 C-7, 139.5 C-9, 140.4 C-4	22.8 C-8	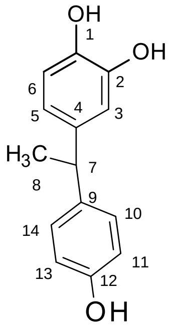
3.89, q, J = 7.2 Hz, 1H, H-7	22.8 C-8, 115.9 C-3, 119.8 C-5, 129.4 C-10/C-14, 139.5 C-9, 140.4 C-4	44.6 C-7	
6.53, dd, J = 1.6 Hz, J= 8.1 Hz, 1H, H-5	44.6 C-7, 115.9 C-3, 144.2 C-1	119.8 C-5	
6.60, d(s), J = 1.6 Hz, 1H, H-3	44.6 C-7, 119.8 C-5, 144.2 C-1, (146.0 C-2)	115.9 C-3	
6.66, d, 1H, H-6	140.4 C-4, (144.2 C-1), 146.0 C-2	116.1 C-6	
6.68, d, J = 8.5 Hz, 2H, H-11/H-13	116.0 C-11/C-13, 139.5 C-9, (156.4 C-12)	116.0 C-11/C-13	
7.00, d, J = 8.5 Hz, 2H, H-10/H-14	44.6 C-7, 129.4 C-10/C-14, 156.4 C-12	129.4 C-10/C-14	
		139.5 C-9	
		140.4 C-4	
		144.2 C-1	
		146.0 C-2	
		156.4 C-12	

Table S3: GC-MS and HPLC-MS data of bisphenol analogues and their products II (*ortho*-hydroxylated at both phenol rings, BP-2xOH) formed during the incubation of *Cupriavidus basilensis* SBUG 290 with BPAP and BPZ in comparison to the corresponding product II_{BPA} of BPA-transformation (Zühlke et al. 2017). Chemical structures and names of products are given in Table S9 (page 15/16)

bisphenol	molecular weight (g moL^{-1})	product	theoretical molecular weight (g moL^{-1})	mass difference between bisphenol and transformation product	HPLC-MS data		Base peak ion (GC-MS) and if applicable number of methylated derivatives[a]
					negative ion mode $[M-H]^-$	positive ion mode $[M+H]^+$	
BPA	228	product II_{BPA}	260	Δ32 g moL^{-1}	*m/z* 259	n.d.	n.d
BPAP	290	product II_{BPAP}	322	Δ32 g moL^{-1}	*m/z* 321	n.d.	n.d.
BPZ	268	product II_{BPZ}	300	Δ32 g moL^{-1}	*m/z* 299	n.d.	2x *m/z* 342 (M^++3CH_3)<br>1x *m/z* 356 (M^++4CH_3)

n.d. not detected; [a]M^++3CH_3 trimethylated- or M^++4CH_3 tetramethylated-derivative, 1-2x refers to number of detected derivatized substances: four hydroxyl groups within the molecule (see Table S 9) would allow up to four methylations and different combinations thereof to be detected by GC-MS

Table S4: GC-MS and HPLC-MS data of bisphenol analogues and their products III (ring fission products, BP-lactone) formed during the incubation of *Cupriavidus basilensis* SBUG 290 with BPAP, BPB, BPE, BPF, and BPZ in comparison to the corresponding product III_{BPA} of BPA-transformation (Zühlke et al. 2017). Chemical structures and names of products are given in Table S9 (page 15/16)

bisphenol	molecular weight (g moL^{-1})	product	theoretical molecular weight (g moL^{-1})	mass difference between bisphenol and transformation product	HPLC-MS data		Base peak ion (GC-MS) and if applicable number of methylated derivatives[a]
					negative ion mode $[M-H]^-$	positive ion mode $[M+H]^+$	
BPA	228	product III_{BPA}	274	Δ46 g moL^{-1}	*m/z* 273	*m/z* 275	1x *m/z* 302 ($M+2CH_3$)
BPAP	290	product III_{BPAP}	336	Δ46 g moL^{-1}	*m/z* 335	m/z 337	n.d.
BPB	242	product III_{BPB}	288	Δ46 g moL^{-1}	*m/z* 287	*m/z* 289	1x *m/z* 302 (M^++1CH_3)<br>1x *m/z* 316 (M^++2CH_3)
BPE	214	product III_{BPE}	260	Δ46 g moL^{-1}	n.d.	*m/z* 261	1x *m/z* 274 (M^++1CH_3)<br>1x *m/z* 288 (M^++2CH_3)
BPF	200	product III_{BPF}	246	Δ46 g moL^{-1}	n.d.	n.d.	n.d.
BPZ	268	product III_{BPZ}	314	Δ46 g moL^{-1}	*m/z* 313	*m/z* 315	1x *m/z* 328 (M^++1CH_3)<br>1x *m/z* 342 (M^++2CH_3)

n.d. not detected; [a]M^++1CH_3 monomethylated- or M^++2CH_3 dimethylated-derivative, 1x refers to number of detected derivatized substances: one hydroxyl and one carboxyl group within the molecule (see Table S 9) would allow up to two methylations and different combinations thereof to be detected by GC-MS

Table S5: NMR data and structure of product III_{BPE} (BPE-lactone) formed during the incubation of *Cupriavidus basilensis* SBUG 290 with BPE

^1H	^1H-^{13}C-correlation	^{13}C	Structure
1.53, d, J = 7.1 Hz, 3H, H-9<br>3.89, q, J = 7.1 Hz, 1H, H-8<br>6.24, s, 1H, H-3<br>6.74 , d, J = 8.4 Hz, 2H, H-12/H14<br>6.85, s, 1H, H-5<br>7.07, d, J = 8.4 Hz, 2H, H-11/H-15	not performed	not performed	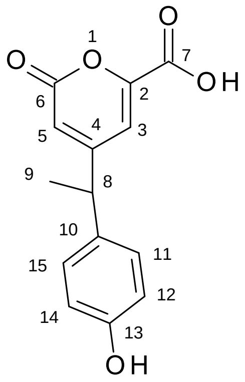

Table S6: GC-MS and HPLC-MS data of bisphenol analogues and their products IV (products with an acetamide substituent, BP-acetamide) formed during the incubation of *Cupriavidus basilensis* SBUG 290 with BPB, BPE, and BPF in comparison to the corresponding product IV_{BPA} of BPA-transformation (Zühlke et al. 2017). Chemical structures and names of products are given in Table S9 (page 15/16)

bisphenol	molecular weight (g moL^{-1})	product	theoretical molecular weight (g moL^{-1})	mass difference between bisphenol and transformation product	HPLC-MS data		Base peak ion (GC-MS) and if applicable number of methylated derivatives[a]
					negative ion mode $[M-H]^-$	positive ion mode $[M+H]^+$	
BPA	228	[1]product IV_{BPA}	285	Δ57 g moL^{-1}	*m/z* 284	*m/z* 286	n.d.
BPB	242	product IV_{BPB}	299	Δ57 g moL^{-1}	*m/z* 298	*m/z* 300	n.d.
BPE	214	product IV_{BPE}	271	Δ57 g moL^{-1}	n.d.	*m/z* 272	*m/z* 271 (M^+)<br>1x *m/z* 285 (M^++1CH_3)
BPF	200	product IV_{BPF}	257	Δ57 g moL^{-1}	n.d.	*m/z* 258	n.d.

n.d. not detected; [a]M^++1CH_3 monomethylated-derivative, 1x refers to number of detected derivatized substances: two hydroxyl groups within the molecule (see Table S 9) would allow up to two methylations and different combinations thereof to be detected by GC-MS

Table S7: NMR data and structure of product IV_{BPE} (BPE-acetamide) formed during the incubation of *Cupriavidus basilensis* SBUG 290 with BPE

^{1}H	^{1}H-^{13}C-correlation	^{13}C	Structure
1.53, d, J = 7.1 Hz, 3H, H-8<br>3.96, q, J = 7.1 Hz, 1H, H-7<br>6.68, m, J = 8.3 Hz, 4H (1s 1H, 1d 1H, 1d 2H), H-3/H-5/H-11/H-13<br>7.01, d, J = 8.3 Hz, 2H + 1H, H-10/H-14<br>7.41, d, J = 8.1 Hz, 1H, H-6	not performed	not performed	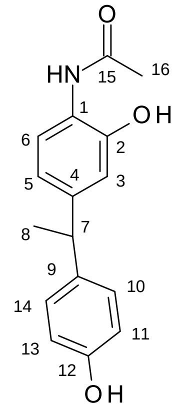

Table S8: GC-MS and HPLC-MS data of the *ortho*-hydroxylated ring fission product VI_{BPZ} (BPZ-lactone-OH) in comparison to the non-hydroxylated ring fission product III_{BPZ} (BPZ-lactone), both formed during the incubation of *Cupriavidus basilensis* SBUG 290 with BPZ. Chemical structures and names of products are given in Table S9 (page 15/16)

product	theoretical molecular weight (g moL^{-1})	mass difference between both transformation products	HPLC-MS data		Base peak ion (GC-MS) and if applicable number of methylated derivatives[a]
			negative ion mode $[M-H]^-$	positive ion mode $[M+H]^+$	
Product III_{BPZ}	314	Δ16 g moL^{-1}	*m/z* 313	*m/z* 315	1x *m/z* 328 (M^++1CH_3)
					1x *m/z* 342 (M^++2CH_3)
Product VI_{BPZ}	330		*m/z* 329	*m/z* 331	1x *m/z* 372 (M^++3CH_3)

[a]M^++1CH_3 monomethylated-, M^++2CH_3 dimethylated- or M^++3CH_3 trimethylated-derivative, 1x refers to number of detected derivatized substances: one or two hydroxyl groups and one carboxyl group within the molecule (see Table S 9) allow two up to three methylations and different combinations thereof to be detected by GC-MS

Table S9: Postulated structure and chemical name of the products formed during the incubation of *Cupriavidus basilensis* SBUG 290 with various bisphenol analogues

Substrate	Product	Postulated structure	Postulated name
BPAP	product I_{BPAP} (BPAP-OH)	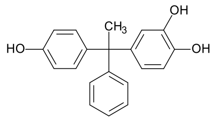	4-[1-(4-hydroxyphenyl)-1-phenylethyl]benzene-1,2-diol
	product II_{BPAP} (BPAP-2xOH)	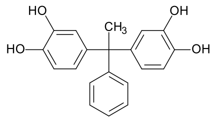	4-[1-(3,4-dihydroxyphenyl)-1-phenylethyl]benzene-1,2-diol
	product III_{BPAP} (BPAP-lactone)	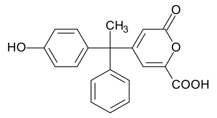	4-[1-(4-hydroxyphenyl)-1-phenylethyl]-6-oxo-6H-pyran-2-carboxylic acid
BPB	product I_{BPB} (BPB-OH)	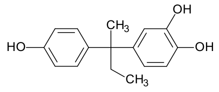	4-[1-(4-hydroxyphenyl)-1-methyl-propyl]benzene-1,2-diol
	product III_{BPB} (BPB-lactone)	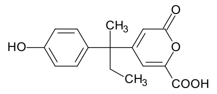	4-[1-(4-hydroxyphenyl)-1-methyl-propyl]-6-oxo-pyran-2-carboxylic acid
	product IV_{BPB} (BPB-acetamide)	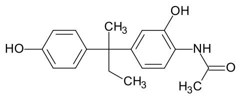	N-[2-hydroxy-4-[1-(4-hydroxyphenyl)-1-methyl-propyl]phenyl]acetamide
	product V_{BPB} (BPB-dimer)	dimer of modified BPB monomers	
BPC	product I_{BPC} (BPC-OH)	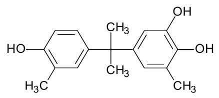	5-[1-(4-hydroxy-3-methyl-phenyl)-1-methyl-ethyl]-3-methyl-benzene-1,2-diol
	product 1_{BPC} (BPC-CH_2OH)	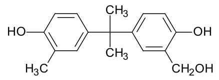	2-(hydroxymethyl)-4-[1-(4-hydroxy-3-methyl-phenyl)-1-methyl-ethyl]-phenol
	product 2_{BPC} (BPC-COOH)	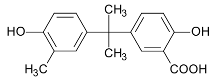	2-hydroxy-5[1-(4-hydroxy-3-methyl-phenyl)-1-methyl-ethyl]benzoic acid
	product 3_{BPC} (BPC-CH_2OH-OH)	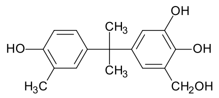	3-(hydroxymethyl)-5-[1-(4-hydroxy-3-methyl-phenyl)-1-methyl-ethyl]benzene-1,2-diol
	product 4_{BPC} (BPC-COOH-OH)	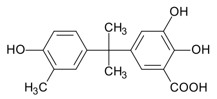	2,3-dihydroxy-5-[1-(4-hydroxy-3-methyl-phenyl)-1-methyl-ethyl]benzoic acid

Table S9 continued

Substrate	Product	Postulated structure	Postulated name
BPE	product I_{BPE} (BPE-OH)	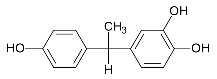	4-[1-(4-hydroxyphenyl)ethyl]benzene-1,2-diol
	product III_{BPE} (BPE-lactone)	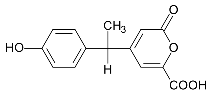	4-[1-(4-hydroxyphenyl)ethyl]-6-oxo-pyran-2-carboxylic acid
	product IV_{BPE} (BPE-acetamide)	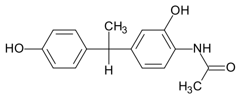	N-[2-hydroxy-4-[1-(4-hydroxyphenyl)ethyl]phenyl]acetamide
BPF	product I_{BPF} (BPF-OH)	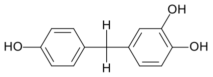	4-[(4-hydroxyphenyl)methyl]benzene-1,2-diol
	product III_{BPF} (BPF-lactone)	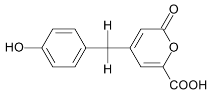	4-[(4-hydroxyphenyl)methyl]-6-oxo-pyran-2-carboxylic acid
	product IV_{BPF} (BPE-acetamide)	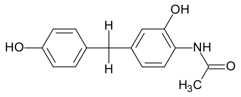	N-[2-hydroxy-4-[(4-hydroxyphenyl)methyl]phenyl]acetamide
BPPH	product 2_{BPPH} (BPPH-COOH)	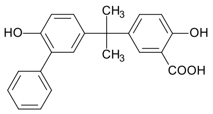	2-hydroxy-5-[1-(4-hydroxy-3-phenyl-phenyl)-1-methyl-ethyl]benzoic acid
	product 5_{BPPH} (BPPH-2xCOOH)	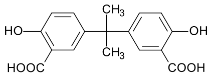	5-[1-(3-carboxy-4-hydroxy-phenyl)-1-methyl-ethyl]-2-hydroxy-benzoic acid
BPZ	product I_{BPZ} (BPZ-OH)	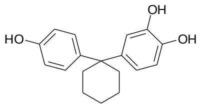	4-[1-(4-hydroxyphenyl)-cyclohexyl]benzene-1,2-diol
	product II_{BPZ} (BPZ-2xOH)	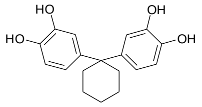	4-[1-(3,4-dihydroxyphenyl)-cyclohexyl]benzene-1,2-diol
	product III_{BPZ} (BPZ-COOH)	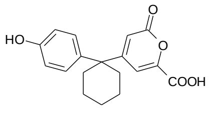	4-[1-(4-hydroxyphenyl)-cyclohexyl]-6-oxo-pyran-2-carboxylic acid
	product VI_{BPZ} (BPZ-COOH-OH)	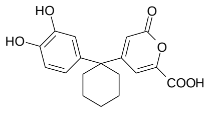	4-[1-(3,4-dihydroxyphenyl)-cyclohexyl]-6-oxo-pyran-2-carboxylic acid

Table S10: List of products formed during incubation of *Cupriavidus basilensis* SBUG 290 with BPAP, BPC, BPZ and BPPH, but not characterized due to their low concentration

Bisphenol	Uncharacterized product	HPLC retention time [min]	UV-Vis absorption maxima [nm]
BPAP	a	7.7	216, 280
	b	7.0	218, 278
	c	6.0	220, 280
BPC	a	9.4	224, 272, 406
BPZ	a	6.9	230, 278
BPPH	a	11.2	216, 232, 314
	b	10.7	220, 246, 320
	c	10.3	222, 250, 320
	d	10.1	222, 248, 318
	e	9.3	222, 248, 320
	f	8.7	238, 314
	g	5.6	222, 280

Table S11: GC-MS and HPLC-MS data of products formed during the incubation of *Cupriavidus basilensis* SBUG 290 with BPC in comparison to product I_{BPA} and BPA (Zühlke et al. 2017). Chemical structure of products is given in Table S9 (page 15/16)

bisphenol	molecular weight (g moL^{-1})	product	theoretical molecular weight (g moL^{-1})	mass difference between bisphenol and transformation product	HPLC-MS data of the transformation products		Base peak ion (GC-MS) and if applicable number of methylated derivatives[b]
					negative ion mode $[M-H]^-$	positive ion mode $[M+H]^+$	
BPA	228	product I_{BPA}	244	Δ16 g moL^{-1}	*m/z* 243	n.d.	3x *m/z* 258 (M^++1CH_3)<br>3x *m/z* 272 (M^++2CH_3)<br>1x *m/z* 286 (M^++3CH_3)
BPC	256	product I_{BPC}	272	Δ16 g moL^{-1}	[a]*m/z* 269	n.d.	*m/z* 272 (M^+)
BPC	256	product 1_{BPC}	272	Δ16 g moL^{-1}	n.d.	n.d.	1 x *m/z* 286 (M^++CH_3)
BPC	256	product 2_{BPC}	286	Δ30 g moL^{-1}	n.d.	*m/z* 287	*m/z* 286 (M^+)<br>*m/z* 300 (M^++1CH_3)
BPC	256	product 3_{BPC}	288	Δ32 g moL^{-1}	[a]*m/z* 285	n.d.	n.d.

n.d. not detected; [a]HPLC-MS analysis detected an *ortho*-quinoid derivative of the product in the negative ion mode; [b]M^++1CH_3 monomethylated-, M^++2CH_3 dimethylated- or M^++3CH_3 trimethylated-derivative, 1-3x refers to number of detected derivatized substances: hydroxyl, hydroxymethyl or carboxyl groups within the molecule (see Table S 9) allow two up to four methylations and different combinations thereof to be detected by GC-MS

Table S 12: NMR data and structure of product I_{BPC} (BPC-OH) formed during the incubation of *Cupriavidus basilensis* SBUG 290 with BPC

^{1}H	^{1}H-^{13}C-Correlation	^{13}C	Structure
1.53, s, 6H, H-8/H-9	31.7, 42.6, 143.4 , 144.1	not performed	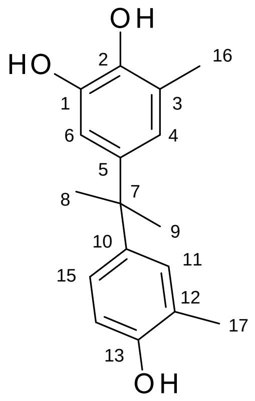
2.12, s, 3H, H-16 or H-17	124.6 , 130.5 , 154.2		
2.14, s, 3H, H-16 or H-17	121.0 , 125.0 , 142.1		
6.46, s, 1H, H-4 or H-6	42.6, 121.0, 142.1, 145.5		
6.47, s, 1H, H-4 or H-6	16.3, 42.6 , 113.1, 142.1		
6.61, d, J = 8.3 Hz, 1H, H-14	124.6, 143.4, 154.2		
6.85, dd, J = 8.2 Hz, J = 2.3 Hz, 1H, H-15	42.6, 130.5, 154.2		
6.89, d(s), 1H, H-11	16.3, 42.6, 126.1, 154.2		
1.53, s, 6H, H-8/H-9	31.7, 42.6, 143.4 , 144.1		

Table S13: NMR data and structure of product 1_{BPC} (BPC-CH_2OH) formed during the incubation of *Cupriavidus basilensis* SBUG 290 with BPC

^{1}H	^{1}H-^{13}C-correlation	^{13}C	structure
1.58, s, 6H, H-8/H-9	31.7 C-8/C-9, 42.6 C-7, 143.5 C-10, 143.6 C-4	31.7 C-8/C-9	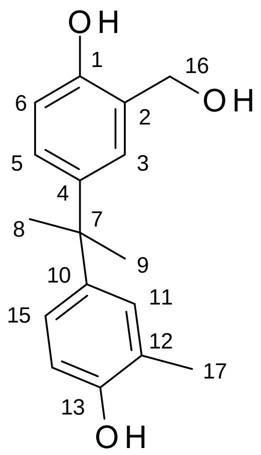
2.11, s, 3H, H-17	125.3 C-12, 130.0 C-11, 154.4 C-13	16.3 C-17	
4.60, s, 2H, H-16	((115.3 C-6)), 127.8 C-3, 154.2 C-1	61.4 C-16	
6.61, d, J = 8.3 Hz, 1H, H-14	((16.3 C-17)), ((42.6 C-7)), 125.3 C-12, (130.0 C-11), 143.5 C-10, (154.4 C-13)	114.8 C-14	
6.65, d, J = 8.4 Hz, 1H, H-6	(61.4 C-16), 127.7 C-2, 143.6 C-4, (154.2 C-1)	115.3 C-6	
6.86, dd, J = 8.3 Hz, J = 2.1 Hz, 1H, H-15	42.6 C-7, (125.3 C-12), 130.0 C-11, 154.4 C-13	125.8 C-15	
6.89, d(s), J = 2.1 Hz, 1H, H-11	16.3 C-17, 42.6 C-7, 125.8 C-15, 154.4 C-13	130.0 C-11	
6.95, dd, J = 8.4 Hz, J = 2.3 Hz, 1H, H-5	42.6 C-7, 127.8 C-3, 154.2 C-1	127.6 C-5	
7.14, d(s), J = 2.3 Hz, 1H, H-3	42.6 C-7, 61.4 C-16, (115.3 C-6), 127.6 C-5, 154.2 C-1	127.8 C-3	
		42.6 C-7	
		125.3 C-12	
		127.7 C-2	
		143.6 C-4	
		143.5 C-10	
		154.2 C-1	
		154.4 C-13	

Table S14: NMR data and structure of product 2_{BPC} (BPC-COOH) formed during the incubation of *Cupriavidus basilensis* SBUG 290 with BPC

^{1}H	^{1}H-^{13}C-Correlations	^{13}C	structure
1.59, s, 6H, H-8/H-9	31.5 C-8/C-9, 42.5 C-7, 142.7 C-10, 143.1 C-5	31.5 C-8/C-9	
2.12, s, 3H, H-17	((115.1 C-14)), 124.9 C-12, 130.3 C-11, 154.3 C-13	16.5 C-17	
6.63, d, J = 8.3 Hz, 1H, H-14	((16.5 C-17)), 124.9 C-12, (130.3 C-11), 142.7 C-10, (154.3 C-13)	115.1 C-14	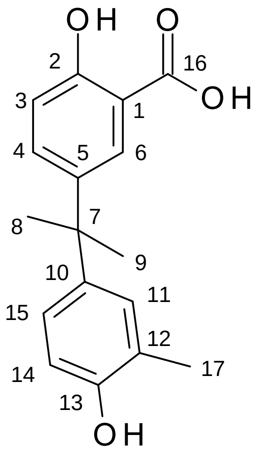
6.75, d, J = 8.6 Hz, 1H, H-3	115.0 C-1, 143.1 C-5, 160.9 C-2, (174.3 C-16)	117.4 C-3	
6.85, dd, J = 8.3 Hz, J = 1.9 Hz, 1H, H-15	42.5 C-7, 130.3 C-11, 154.3 C-13	125.9 C-15	
6.90, d(s), J = 1.9 Hz, 1H, H-11	16.5 C-17, 42.5 C-7, 125.9 C-15, 154.3 C-13	130.3 C-11	
7.20, dd, J = 8.6 Hz, J = 1.8 Hz, 1H, H-4	42.5 C-7, 128.8 C-6, 160.9 C-2	135.2 C-4	
7.80, d(s), J = 1.8 Hz, 1H, H-6	42.5 C-7, 135.2 C-4, 160.9 C-2, 174.3 C-16	128.8 C-6	
		42.5 C-7	
		115.0 C-1	
		124.9 C-12	
		142.7 C-10	
		143.1 C-5	
		154.3 C-13	
		160.9 C-2	
		174.3 C-16	

Table S 15: NMR data and structure of BPPH

^{1}H	^{1}H-^{13}C-correlation	^{13}C	structure
1.68, s, 6H, H-8/H-9	31.7 C-8/C-9, 42.7 C-7, 143.8 C-4	31.7 C-8/C-9	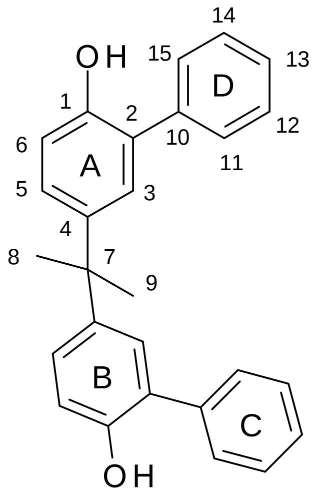
6.82, d, J = 8.5 Hz, 2H, H-6	129.3 C-2, 143.8 C-4, (152.9 C-1)	116.6 C-6	
7.08, m, J = 8.5 Hz, J = 2.3 Hz, 2H, H-5	42.7 C-7, ((116.6 C-6)), 130.2 C-3, 152.9 C-1	127.9 C-5	
7.15, d(s), J = 2.3 Hz, 2H, H-3	42.7 C-7, ((116.6 C-6)), 127.9 C-5, 140.7 C-10, 152.9 C-1	130.2 C-3	
7.26, t, J = 7.3 Hz, 2H, H-13	130.4 C-11/C-15, ((140.7 C-10))	127.5 C-13	
7.36, dd, J = 7.3 Hz, J = 7.5 Hz, 4H, H-12/H-14	129.0 C-12/C-14, 140.7 C-10	129.0 C-12/C-14	
7.50, d, J = 7.5 Hz, 4H, H-11/H-15	127.5 C-13, 130.4 C-11/C-15	130.4 C-11/C-15	
		42.7 C-7	
		129.3 C-2	
		140.7 C-10	
		143.8 C-4	
		152.9 C-1	

Table S 16: NMR data and structure of product 2_{BPPH} (BPPH-COOH) formed during the incubation of *Cupriavidus basilensis* SBUG 290 with BPPH

^{1}H	^{13}C	Rings	structure
1.67, s, 6H, H-9/H-10	31.4 C-9/C-10		
6.83, d, J = 8.6 Hz, 1H, H-15	116.7 C-15	B	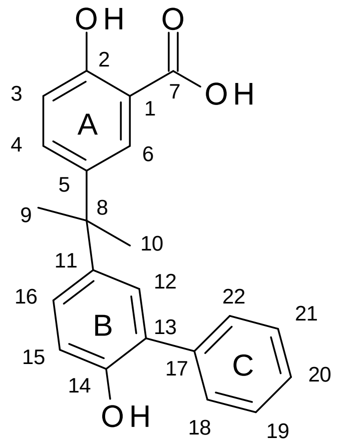
6.84, d, J = 8,8 Hz, 1H, H-3	117.8 C-3	A	
7.03, m, J = 8.6 Hz, J = 2.5 Hz, 1H, H-16	127.8 C-16	B	
7.11, d(s), J = 2.5 Hz, 1H, H-12	130.0 C-12	B	
7.27, t, J = 7.4 Hz, 1H, H-20	127.6 C-20	C	
7.34, m, J = 8.8 Hz, J = 2.5 Hz, 1H, H-4	135.8 C-4	A	
7.37, dd, J = 7.4 Hz, J = 7.9 Hz, 2H, H-19/H-21	129.0 C-19/C-21	C	
7.49, d, J = 7.9 Hz, 2H, H-18/H-22	130.4 C-18/C-22	C	
7.86, d(s), J = 2.5 Hz, 1H, H-6	128.7 C-6	A	
	42.7 C-8		
	113.2 C-1	A	
	128.7		
	129.4		
	140.5		
	143.0		
	153.1 C-14	B	
	161.0 C-2	A	
	173.7 C-7		

Table S17: Comparison of NMR data and structure of product 5_{BPPH} (BPPH-2xCOOH) and product 2_{BPPH} (BPPH-COOH) formed during the incubation of *Cupriavidus basilensis* SBUG 290 with BPPH as well as product 2_{BPC} (BPC-COOH) formed during the incubation of *Cupriavidus basilensis* SBUG 290 with BPC

product 5_{BPPH}	product 2_{BPC}	product 2_{BPPH}
^{1}H NMR (ring A)	^{1}H NMR (ring A)	^{1}H NMR (ring A)
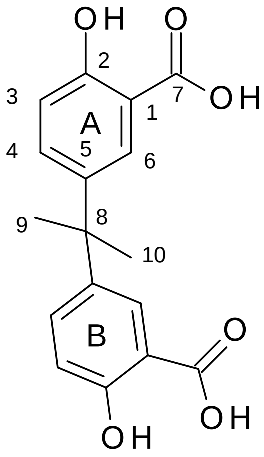	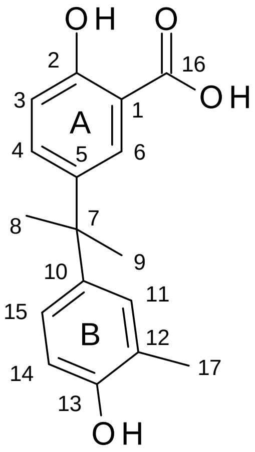	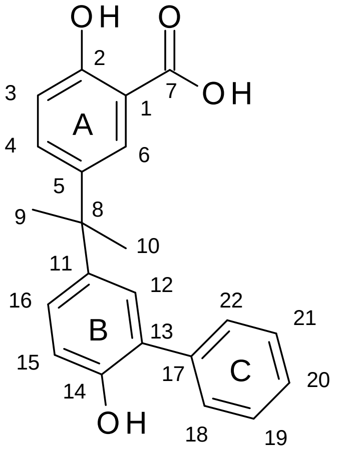
6.83, d, J = 8,7 Hz, 1H, H-3<br>7.28, m, J = 8.7 Hz, J = 2.5 Hz, 1H, H-4<br>7.86, d(s), J = 2.5 Hz, 1H, H-6	6.75, d, J = 8.6 Hz, 1H, H-3<br>7.20, dd, J = 8.6 Hz, J = 1.8 Hz, 1H, H-4<br>7.80, d(s), J = 1.8 Hz, 1H, H-6	6.84, d, J = 8,8 Hz, 1H, H-3<br>7.34, m, J = 8.8 Hz, J = 2.5 Hz, 1H, H-4<br>7.86, d(s), J = 2.5 Hz, 1H, H-6

Table S18: GC-MS and HPLC-MS data of the two major products formed during the incubation of *Cupriavidus basilensis* SBUG 290 with BPPH. Chemical structures and names of products are given in Table S9 (page 15/16)

bisphenol	molecular weight (g moL^{-1})	product	theoretical molecular weight (g moL^{-1})	mass difference between bisphenol and transformation product	HPLC-MS data of the transformation products		Base peak ion (GC-MS) and if applicable number of methylated derivatives
					negative ion mode $[M-H]^-$	positive ion mode $[M+H]^+$	
BPPH	380	product 2_{BPPH}	348	Δ32	*m/z* 347	*m/z* 349	n.d.
BPPH	380	product 5_{BPPH}	316	Δ64	*m/z* 315	*m/z* 317	n.d.

n.d. not detected

REFERENCES

Zühlke M-K, Schlüter R, Mikolasch A, Zühlke D, Giersberg M, Schindler H, Henning A-K, Frenzel H, Hammer E, Lalk M (2017) Biotransformation and reduction of estrogenicity of bisphenol A by the biphenyl-degrading *Cupriavidus basilensis*. Appl Microbiol Biotechnol 101(9):3743-3758
